# Cellular, molecular, and clinical mechanisms of action of deep brain stimulation—a systematic review on established indications and outlook on future developments

**DOI:** 10.15252/emmm.201809575

**Published:** 2019-03-12

**Authors:** Martin Jakobs, Anton Fomenko, Andres M Lozano, Karl L Kiening

**Affiliations:** ^1^ Department of Neurosurgery University Hospital Heidelberg Heidelberg Germany; ^2^ Division of Neurosurgery Toronto Western Hospital University Health Network Toronto ON Canada

**Keywords:** circuitopathies, deep brain stimulation, movement disorders, neuromodulation, Parkinson's disease, Neuroscience

## Abstract

Deep brain stimulation (DBS) has been successfully used to treat movement disorders, such as Parkinson's disease, for more than 25 years and heralded the advent of electrical neuromodulation to treat diseases with dysregulated neuronal circuits. DBS is now superseding ablative techniques, such as stereotactic radiofrequency lesions. While serendipity has played a role in developing DBS as a therapy, research during the past two decades has shown that electrical neuromodulation is far more than a functional lesion that can be switched on and off. This understanding broadens the field to enable new types of stimulation, clinical indications, and research. This review highlights the complex effects of DBS from the single cell to the neuronal network. Specifically, we examine the electrical, cellular, molecular, and neurochemical mechanisms of DBS as applied to Parkinson's disease and other emerging applications.

**Glossary**
Action potential (AP): A.k.a. nerve impulse or spike: a propagation of electrical depolarization along the length of a neuron's axon, mainly modulated by voltage‐gated on channels. Otherwise known as a nerve impulse.
Afferent: Nerve fibers (axons) that arrive at a certain brain region and convey information from other brain regions via synapses on the dendrites of that target neuron.
Anhedonia: A loss of interest or reduced ability to experience pleasure. A core component of several depression‐related disorders.
Antidromic: Propagation of an action potential along an axon in the opposite direction of usual (orthodromic) conduction. Antidromic excitation of a nerve refers to travel of an electrical signal away from axon terminals and toward the cell body.
Bradykinesia: A.k.a. slowing of movement: Along with tremor and rigidity, bradykinesia is a cardinal symptom of certain neurodegenerative disorders such as Parkinson's disease. Bradykinesia can sometimes also be a side effect of certain medications.
Cyclic voltammetry: An electrochemical analytical technique that allows monitoring of neurotransmitter concentrations by probing different brain regions with an electrode. Neurotransmitters detected using this technique include dopamine, serotonin, and norepinephrine.
Deafferentation: A condition resulting from the elimination or interruption of nerve fibers (afferents) that carry sensory information to the brain. Potential causes of this condition are disease, injury, or in an experimental context, an intentional lesion caused to study resulting effects on the peripheral and central nervous system.
Deep brain stimulation (DBS): A surgical procedure involving implantation of an implantable pulse generator (IPG), leads, and electrode(s) to deliver chronic electrical stimulation of brain region(s). DBS is used either for therapy for disorders affecting the nervous system, especially movement disorders, and has been approved in most countries for the treatment of Parkinson's disease, essential tremor, and dystonia. Experimental applications include other neurodegenerative conditions such as Alzheimer's disease, as well as psychiatric disorders such as depression.
Depolarization block: An electrical event within a neural membrane caused by sustained input currents, especially at strong intensities. For example, during seizures, sustained firing of neurons may lead to a depolarization block mediated by persistent sodium currents, which limits the maximum firing rate of the cells.
Dyskinesia: An abnormal, involuntary, repetitive movement of a limb or part of the body, for example, a twitch. In context of Parkinson's disease, dyskinesia is a common side effect of long‐term use of antiparkinsonian dopaminergic medications, such as levodopa.
Dystonia: A movement disorder in which sustained, involuntary muscle contractions cause painful and abnormally contorted body or limb positions. Dystonia can be either congenital or acquired and is one of the common indications for DBS treatment, with a common target being the globus pallidus interna.
Efferent: Nerve fibers (axons) that exit from a certain brain region and convey information from the target neurons via synapses on the dendrites of other brain regions.
Entorhinal cortex (EC): A cortical brain region located inferior to the rhinal sulcus within the medial temporal lobe. Purported to serve as a hub for memory‐related pathways and a site of experimental DBS for Alzheimer's disease, especially in animal models.
Implantable pulse generator (IPG): A small pacemaker‐like implantable medical device which both controls and provides electrical power necessary for a DBS system. Typically implanted under the chest skin, the IPG contains either a rechargeable or single‐use battery along with one or more output cables that connect to the stimulating electrodes.
Magnetoencephalography (MEG): A non‐invasive functional brain imaging technique that detects the minuscule electromagnetic fluctuations emitted by the human brain, allowing for monitoring of awake brain activity in multiple cortical brain regions.
Orthodromic: The typical direction of an action potential's propagation, namely away from the neuron cell body, or soma, and toward the axon terminals. Its opposite is antidromic.
Pallidotomy: A surgical procedure involving targeted destruction of part of the globus pallidus internus. Sometimes used as an alternative to DBS, this procedure is more commonly used to treat severe cases of levodopa‐induced dyskinesia, a common side effect of antiparkinsonian medications.
Positron emission tomography (PET): A functional brain imaging technique that uses intravenously injected tracers that emit positrons (gamma radiation) to assess metabolic activity. Different tracers are available for different organs or conditions. Gamma radiation is detected over time (dynamic) or at a given point in time after injection of the tracer (static). Results are usually visualized with a three‐dimensional CT scan.
Stereotactic surgery: Stereotactic surgery is a minimally invasive form of neurosurgery that utilizes a virtual three‐dimensional coordinate system to localize small and often deep‐seated brain region so be treated. A stereotactic frame may be placed on the patient head which is utilized as a base for imaging and to anchor the surgical device to bring electrodes, probes, or catheters to this target area. So‐called frameless systems utilize head‐mounted devices to reach the deep‐seated target regions.
Stimulating electrode: A thin metallic conductor of electricity that is implanted within the brain at the desired stimulation target. Typically insulated along most of their length, DBS electrodes contain small uninsulated segments near the stimulating tip. Some DBS systems allow for selection of bipolar or unipolar electrode channel configurations depending on which segments are chosen to conduct electrical current.
Striatum: A collection of nuclei within the basal ganglia comprising the caudate, putamen, and nucleus accumbens. A striped appearance on brain imaging explains this region's etymology from the Latin “striped”.
Subcortical: Located within the white matter tracts below the cerebral cortex.
Subthalamic nucleus (STN): The STN is a small paired lens‐shaped nucleus situated inferior to the thalamus. An important coordinator of the output flow from the basal ganglia, the STN has been found to contain “pacemaker neurons” that have spontaneous oscillating and synchronous activity, potentially related to the resting tremor found in Parkinson's patients. Accordingly, the STN is a common site for therapeutic DBS for this disease. The STN is composed of a motor region which is targeted in DBS surgery, but also contains parts connected to the limbic system and to the associative system. Both regions are thought to be avoided during DBS surgery as stimulation of these areas evokes side effects like psychiatric symptoms (i.e., depression/mania for the limbic part) or double vision (for the associative part).
Thalamotomy: A surgical procedure involving targeted destruction of part of the thalamus, a key brain region that relays sensorimotor information to the cerebral cortex. Introduced in the 1950s, thalamotomies are used to alleviate the tremor symptoms of several movement disorders such as Parkinson's disease and essential tremor. Nowadays, this procedure can be performed via traditional surgery or noninvasively using high‐intensity focused ultrasound thalamotomy.
Voltammetry: An electroanalytical method to identify the substrates of sample by analyzing its unique conductive properties.

## Introduction

**Figure 1:**
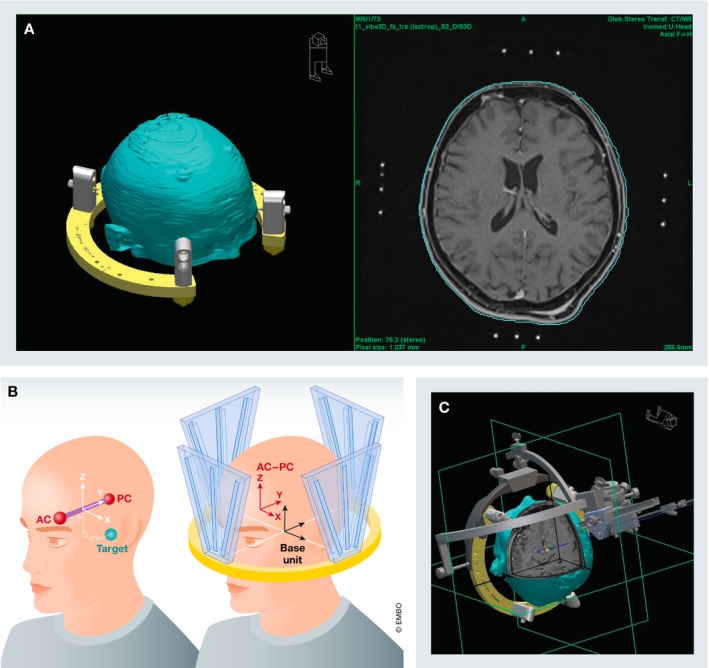
DBS procedure overview DBS procedure: (A) A stereotactic frame is placed on the patient head and fixated with four pins to the outer table of the skull (left). The frame is necessary to acquire stereotactic images (right). (B) A localizer box is placed on the frame (right). The localizer box generates a coordinate system within which each spot can be described using coordinates for laterality (*x*‐axis) anterior–posterior position (*y*‐axis) and superior–inferior position (*z*‐axis). These coordinates are calculated in reference to the line connecting the anterior and posterior border of the third ventricle in the midline (AC‐PC line: anterior commissure—posterior commissure). (C) A stereotactic arc is mounted on the frame set for the calculated target coordinates to implant the electrode in the desired are via a burr hole trephination.

**Figure 2:**
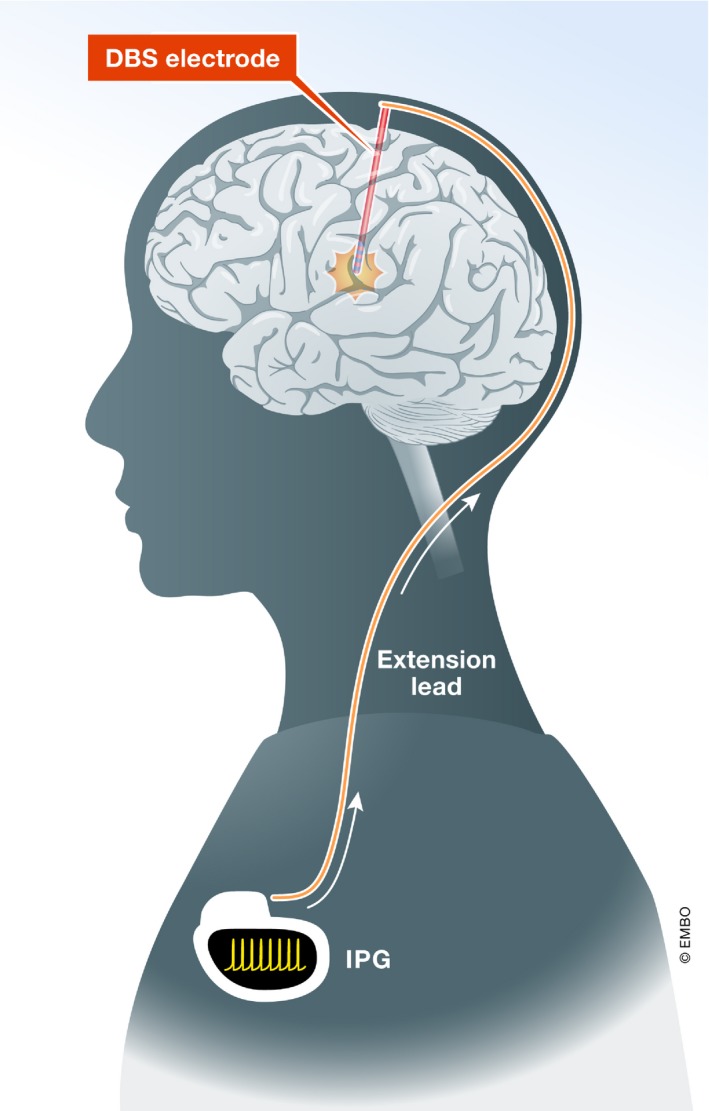
DBS system used for clinical applications Clinically used DBS system: The brain electrode delivers the electrical current for therapeutic purposes. The extension lead connects the brain electrode to the neurostimulator (internal pulse generator, IPG), the implanted power source.

Deep brain stimulation (DBS) has been an established neurosurgical procedure since the 1990s, and more than 160,000 patients worldwide have been operated (Fig 1). During stereotactic surgery, electrodes are implanted in the subcortical nuclei or fiber bundles of the brain, in order to apply chronic low electric currents to therapeutically alter neural function. DBS is an established treatment for movement disorders such as Parkinson's disease (PD; Brown *et al*, 1999; Schuepbach *et al*, 2013), several forms of tremor (Hubble *et al*, 1996), and dystonia (Kumar *et al*, 1999). Other clinical indications are treatment‐resistant epilepsy (Fisher *et al*, 2010) and obsessive–compulsive disorder (OCD; Greenberg *et al*, 2006). Before the advent of DBS, movement disorder surgery encompassed ablative techniques using mostly radiofrequency lesions. Stereotactic lesions in the thalamus (“thalamotomy”; Hassler, 1972) and the pallidum (“pallidotomy”; Spiegel & Wycis, 1952) were commonly used, especially before the introduction of levodopa medication to treat PD. To predict the effects of lesioning prior to a thalamotomy in the ventral intermediate (Vim) nucleus of the thalamus, surgeons used high‐frequency stimulation, which yielded a sustained and immediately reversible effect on tremor (Benabid *et al*, 1996). Thus, the idea to alter the function of neuronal tissue with electricity rather than to destroy it was born. This discovery led to the development of fully implantable DBS systems with dual electrodes connected to an implantable pulse generator (IPG) similar to a cardiac pacemaker to deliver long‐term and continuous therapy (Fig 2).

DBS is nowadays part of a class of treatments known as neuromodulation, which modifies neural function through application of electrical currents. The effects are for the most part immediate, reversible, adaptable, and titratable without permanently damaging neural tissue. Additionally, contrary to lesioning techniques, DBS can be applied bilaterally without causing severe side effects in movement disorder surgery.

The observation that high‐frequency electric stimulation caused the same effects as ablative techniques led to the assumption that DBS inhibits neuronal activity of the stimulated nucleus, causing a functional lesion (Johnson & Vitek, 2011) via depolarization blocks (Meissner *et al*, 2005; McIntyre & Anderson, 2016). While functional inactivation of neurons and depolarization blocks may contribute to the overall effect, further research revealed that the effects of DBS on the cellular, electrical, molecular, and network level by far surpass the effects of lesions. The current hypothesis is that pathologically altered and dysfunctional neuronal circuits, or circuitopathies (Lozano & Lipsman, 2013), are treatable with DBS and that electrical stimulation can alter these neuronal circuits back to a more physiological state (Dostrovsky & Lozano, 2002; Gubellini *et al*, 2009; Johnson & Vitek, 2011; McIntyre & Anderson, 2016).

In this review, we will examine the immediate, short‐term, and long‐term effects of DBS on a cellular, network, and molecular level and highlight investigational and new indications for DBS. A comprehensive literature search was performed and included original articles on *in vitro*,* in vivo,* and clinical research and selected review articles.

## Electrical effects at the stimulation target

The electrical excitability of neural tissue around the electrode and the effects of stimulation are determined by several factors: (i) the proportion of gray and white matter structures (i.e., cell bodies or axons; Nowak & Bullier, 1998; Ranck, 1975; Dostrovsky & Lozano, 2002; Udupa & Chen, 2015); (ii) the type of ion channels on the cell membrane of a soma or axon (Hoang *et al*, 2017); (iii) the diameter and degree of myelination of axons (Ranck, 1975; Nowak & Bullier, 1998; Gubellini *et al*, 2009; Carron *et al*, 2013); (iv) the orientation of axons in relation to the electrode (Ranck, 1975; Gubellini *et al*, 2009); (v) the distance of the stimulated structure to the electrode (Deniau *et al*, 2010); and (vi) the microenvironment (astrocytes and microglia; Song *et al*, 2013; Reddy & Lozano, 2017).

Furthermore, excitability is not only influenced by the three‐dimensional space and the position of the electrode, but also influenced by temporal aspects of stimulation as some effects of DBS happen within seconds, while others can take weeks to establish (Song *et al*, 2013; Reddy & Lozano, 2017). Lastly, the parameters of the stimulation itself (single pulse or continuous stimulation, amplitude, voltage, polarity, frequency, pulse width, pulse shape, rhythm) can vary and cause diverse electrical effects (Anderson *et al*, 2004; Kuncel & Grill, 2004; Montgomery & Gale, 2008; Carron *et al*, 2013). In summary, the electrical effects of clinically applied DBS are strongly influenced by the anisotropic nature of the tissue at the target site in relation to the electrode and can therefore cause heterogeneous electrophysiological, structural, molecular, and cellular reactions.

**Figure 3:**
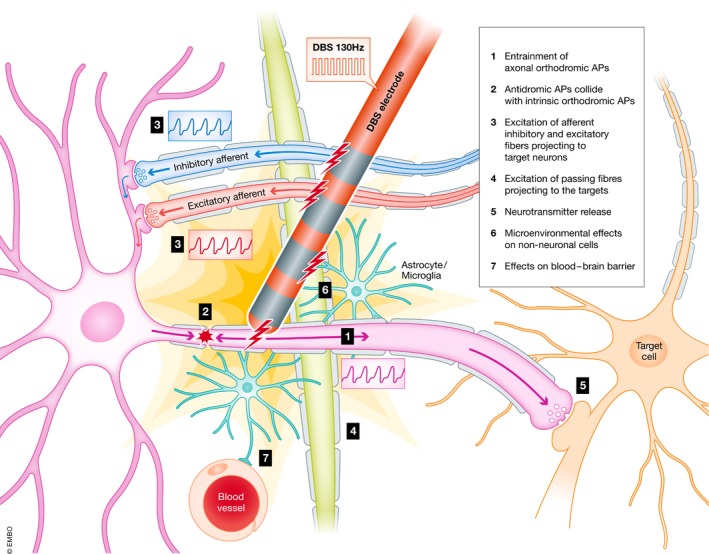
Electrical effects of DBS Electrical effects of DBS: The DBS electrode is implanted in a selected target region. High‐frequency stimulation causes ortho‐ and antidromic axonal action potentials. Somatic action potentials are for the most part blocked from passing through the axon to the synapse. Afferent fibers projecting to the target region and passing fibers of other brain regions are entrained by the frequency of the stimulation. Non‐neural tissue (i.e., astrocytes, microglia, endothelial cells) located in the volume of tissue activated (VTA) is also affected by DBS. These heterogeneous effects combine to irregular neurotransmitter release (“postsynaptic noise”). Pathological neurocircuits are disrupted and moved to a more beneficial state.

**Figure 4:**
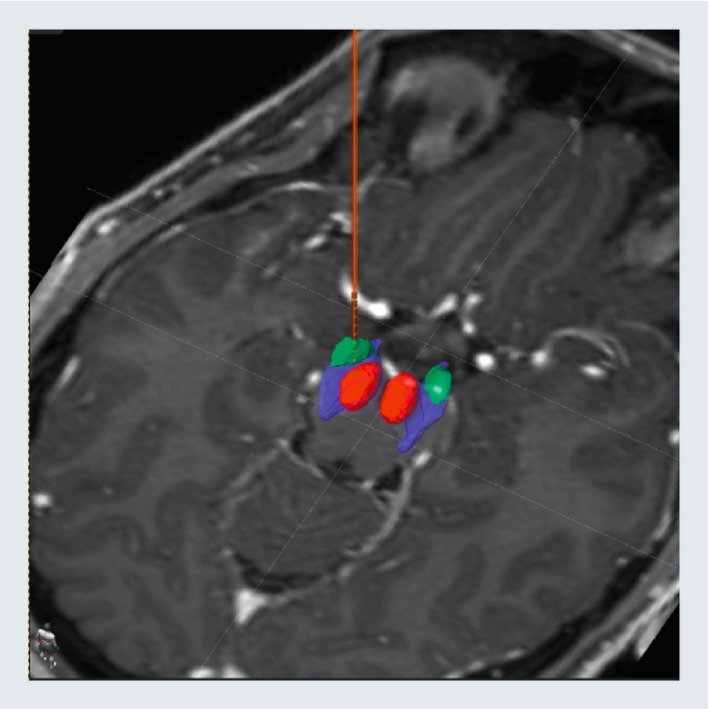
Intraoperative DBS electrode targeting STN‐DBS: DBS electrode implanted in the left dorsolateral subthalamic nucleus (green) in relation to the substantia nigra (blue) and the red nucleus (red).

Although the electrical effects of clinically applied DBS cannot be generalized, several principal cellular effects have been consistently observed (Fig 3). Since stimulation of the subthalamic nucleus (STN) for PD has been the best studied application in experiments, the local and network effects of stimulation will be presented in the context of treating PD (see Fig 4). Stimulation at other targets for other indications may underlie different principles or varying contributions of these principles.

Standard clinically effective STN‐DBS settings comprise the tonic continuous application of monophasic cathodic impulses at a frequency of 130 Hz and a pulse width of 60 μs at an amplitude of 0.8–2.0 mA. Chronaxie and rheobase are two means to describe electrical excitability of neurons and their different components. Rheobase is the amount of current (of infinite duration) necessary to depolarize a neuron and cause an action potential (AP; Nowak & Bullier, 1998; Dostrovsky & Lozano, 2002). Compared to cell bodies, axons have a lower rheobase and therefore show higher levels of excitability (Nowak & Bullier, 1998). Chronaxie is the amount of time needed for a stimulus double the strength of the rheobase to evoke an AP in a neuron (Holsheimer *et al*, 2000). Chronaxies for neuronal cell bodies and dendrites are much longer (1–10 ms) compared to axons (Ranck, 1975), especially myelinated axons with a larger diameter, which have chronaxies between 30 and 300 μs (Holsheimer *et al*, 2000).

Since a single DBS pulse can influence neuronal activities for several milliseconds—7–8 ms in some cases—frequencies with a lesser inter‐stimulus interval (> 125 Hz) will prevent a neuron from fully returning to its base activity (Baker *et al*, 2002; Montgomery & Gale, 2008). It is largely believed that clinically effective high‐frequency DBS preferentially acts on large myelinated axons, depolarizes them, and causes APs (Montgomery & Gale, 2008; Johnson & Vitek, 2011) roughly at the frequency of the stimulation by opening voltage‐gated sodium channels (McIntyre & Anderson, 2016). APs have been shown to travel along a stimulated axon in orthodromic fashion toward the synapse as well as in antidromic fashion toward the soma (Li *et al*, 2007; Agnesi *et al*, 2013; Carron *et al*, 2013).

Orthodromic APs can then trigger neurotransmitter release at the synapse and postsynaptic potentials in the efferent target neurons (Shen *et al*, 2003). Thus, most of the efferent STN axons fire APs in accordance with the frequency of the DBS (Johnson & Vitek, 2011; Humphries & Gurney, 2012) once the stimulation frequency is roughly twice as high as their intrinsic firing frequency (McIntyre & Anderson, 2016). However, the generation and propagation of APs can vary during long‐term stimulation (McIntyre & Anderson, 2016). As the volume of tissue activated (VTA) by clinical DBS does not only contain efferent STN axons but also afferent fibers projecting to STN dendrites and cell bodies, inhibitory (GABAergic) and excitatory (glutamatergic) afferent fibers can be activated and act on the STN cell body itself (Dostrovsky *et al*, 2000; Anderson *et al*, 2004; Gradinaru *et al*, 2009; Chiken & Nambu, 2013).

Cell bodies activated by afferent fibers can generate orthodromic APs that have been shown to collide with antidromic APs generated by axonal stimulation. This phenomenon partially cancels out the intrinsic firing pattern of STN neurons (Magarios‐Ascone *et al*, 2002; Meissner *et al*, 2005). When antidromic APs reach the soma, they initially activate it before constant AP influx overrides the refractory period and prevents the neuron from firing in its previous pattern (Johnson & Vitek, 2011). Two types of reactions have been observed in the soma of STN neurons during stimulation: Type I responses incorporate short phases of depolarization before returning to baseline. Type II responses show a prolonged period of depolarization with or without firing spikes of APs (Shen *et al*, 2003; Anderson *et al*, 2004). This constant trend toward depolarization of cell bodies was thought to be a stable depolarization block comparable to the effects of lesioning techniques (Benazzouz & Hallett, 2000; Beurrier *et al*, 2001; Kringelbach *et al*, 2007b; Hamani *et al*, 2017) to silence the targeted cells.

However, it has been shown that depolarization blocks *per se* are unsustainable states. Cells regain the ability to repolarize over time and fire intrinsic spikes of APs (Ammari *et al*, 2011). Intermittent firing was observed in tonic or burst patterns before the cell enters a phase of depolarization block again. The intrinsic APs have lower amplitudes, but can still evoke neurotransmitter release at the synapse (Florence *et al*, 2009; Ammari *et al*, 2011). Even constantly depolarized membranes in a silenced cell are capable of releasing smaller amounts of neurotransmitters. Depolarization blocks lead to inactivation of voltage‐gated sodium ion channels and accumulation of extracellular potassium (Shin *et al*, 2007). This helps to maintain the resting membrane potential, but has the potential to disturb the conventional mechanisms of repolarization which favors lasting neuronal activity (Florence *et al*, 2009). Over time, Na^+^/K^+^ ATPases ultimately repolarize the cell and enable neuronal firing again. Overall, the firing rate of STN cell bodies is reduced with high‐frequency DBS (Degos, 2005). In summary, DBS therapy seems to inhibit cell bodies and reduce their firing rate while it excites axons and increases their AP frequency.

Astrocytes are another important player in the ion and neurotransmitter microenvironment in the VTA. These glial cells release neurotransmitters and neuromodulators that influence neurons, which might contribute to the therapeutic long‐term effect of DBS (Fenoy *et al*, 2014). Electrical stimulation can activate astrocytes via calcium ion influx, and activated astrocytes have the capability to release glutamate and adenosine and increase the probability of neurotransmitter release from excitatory or inhibitory presynaptic terminals (Sasaki *et al*, 2011). This was shown in *in vitro* experiments: When neuronal synaptic activity was blocked in the thalamus, vesicular glutamate release was still found via astrocytes (Tawfik *et al*, 2010). Furthermore, astrocytes will shift potassium into the extracellular space and therefore contribute to a microenvironment that supports depolarization block‐like states (Sasaki *et al*, 2011; Barat *et al*, 2012; Sauleau *et al*, 2016). The change in the microenvironment of non‐neural cells might lead to prolonged plasticity‐associated effects even when stimulation is turned off. Although there is some *in vitro* evidence that astrocytes may be actively involved in the effects and mechanisms of DBS, no clinical *in vivo* studies have so far been able to prove this theory.

In summary, DBS seems to uncouple STN neurons from its axons and cause a functional deafferentation from both efferent and afferent structures (Dejean *et al*, 2009; Moran *et al*, 2011). Different factors may contribute to the spike activity of inhibited STN neurons: intermittent repolarization during depolarization blocks or preponderance of activation of excitatory afferent projections to the STN neurons. The efferent synapse, however, will not at all times be entrained by the frequency of the stimulation or be completely silenced. Considering the temporospatial aspects of DBS, not all target cells in the VTA may act in complete synchronicity. Some cells may be silenced, and others will be entrained in tonic firing or fire in intermittent bursts. Overall, the axons of the stimulated target are “hijacked” by high‐frequency stimulation, and a new firing pattern is encoded (Benazzouz & Hallett, 2000; Dostrovsky & Lozano, 2002) into the circuit that overwrites the pathological firing pattern in circuitopathies. The collective intrinsic spiking generated by the soma in tonic or burst fashion will cause irregular synaptic and injection of noise in the STN efferents and target neurons (Anderson *et al*, 2004; Moss *et al*, 2004; Hamani *et al*, 2012).

## Electrical effects in the neuronal network

Owing to practical and ethical considerations, human studies on the electrical effects of DBS are most often limited to a single target as the standard of clinical application. These studies either encompass intraoperative measurements via microelectrode recording and microstimulation or recordings from temporarily externalized electrodes after implantation. Multi‐target studies are usually based on *in vivo* animal experiments or *in vitro* brain slices. Most of the knowledge about network effects therefore comes from experimental animal studies.

**Figure 5:**
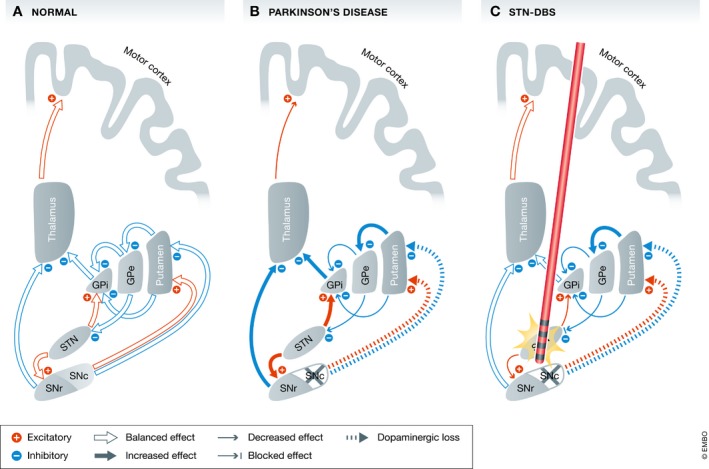
Effects of Parkinson's disease and DBS on basal ganglia networks Basal ganglia models: (A) normal state with physiological balanced output to the motor cortex (B) in Parkinson's disease the dopaminergic loss in the SNc leads to widespread excitatory and inhibitory changes. The STN receives fewer inhibitory signals from the GPe and therefore sends greater excitatory signals to the main basal ganglia output structures (GPi and SNr). The thalamus is inhibited and sends fewer excitatory information to the motor cortex resulting in rigidity and bradykinesia (C) with STN‐DBS the nucleus is uncoupled from its afferents. The excitatory output to the GPi and SNr is reduced which in turn send a more balanced signal the motor cortex via the thalamus resulting in reduced rigidity and bradykinesia. [This model is simplified and leaves the hyperdirect pathway from the motor cortex to the STN and the loop between STN and the pedunculopontine nucleus (PPN)]. GPe: globus pallidus pars externus; GPi: globus pallidus pars internus; SNc: substantia nigra pars compacts; SNr: substantia nigra pars reticulata; and STN: subthalamic nucleus.

The basal ganglia network in healthy individuals entails several neural circuits and both excitatory and inhibitory connections, the correct balance of which enables the appropriate and correct amount of movement (Fig 5A). As a circuitopathy, PD is characterized by the loss of dopaminergic neurons in the substantia nigra pars compacta (SNc) associated with misfolded α‐synuclein proteins and the formation of Lewy bodies (Kalia *et al*, 2013). This dopaminergic loss leads to various and widespread changes in the basal ganglia circuits and cause the main motor symptoms of PD (Fig 5B): bradykinesia, rigidity, and resting tremor. In this pathologically disrupted neural circuit, the electric and functional uncoupling of stimulated STN neurons inhibits the influence of afferent nerves (globus pallidus externus (GPe; Fig 5C), SNc, and the motor cortex through the hyperdirect pathway (Nambu *et al*, 1996). Uncoupling the STN from the SNc makes patients with STN‐DBS less dependent on endogenous and exogenous dopamine.

The main output structures for glutamatergic STN neurons are the globus pallidus pars internus (GPi) and the substantia nigra pars reticulata (SNr; Fig 5A). High‐frequency DBS has been shown to alter neuronal activity in all parts of the basal ganglia, including its main release station within the thalamus (Dorval *et al*, 2008). In the GPi, STN‐DBS causes significant excitation and inhibition in different neurons, while the firing frequency is strongly entrained by the DBS frequency (Hahn & McIntyre, 2010). The GPi showed less activity under STN‐DBS (Hashimoto *et al*, 2003) with increased thalamocortical activity (Fig 5C). STN‐DBS appears to overwrite one form of pathologic activity with an artificially created “noisy” activity, which is less detrimental to the motor circuit.

The pathological basal ganglia network activity in PD is represented by increased synchrony and rhythmicity of neural activity and oscillations. Neural oscillations can be subdivided into different frequency bands: δ (delta: 1–4 Hz), θ (theta: 4–7 Hz), α (alpha: 7–13 Hz), β (beta: 14–30 Hz), and χ (gamma: 30–100 Hz; Başar *et al*, 2013). Theta and especially beta oscillations are thought to be antikinetic (Brown & Williams, 2005) and physiologically maintain the status quo of the resting network (Johnson & Vitek, 2011). Gamma oscillations seem to be associated with hyperkinetic states before and during movements, but also with dyskinetic hypermobility in PD (Brown & Williams, 2005; Udupa & Chen, 2015). Overall, PD patients show an increase in beta and a reduction in gamma oscillations (Silberstein *et al*, 2003; Kühn *et al*, 2006) in most basal ganglia structures, such as the striatum, STN, pallidum, and the motor cortex, which correlates with states of bradykinesia and rigidity (Başar *et al*, 2013; Little & Brown, 2014). Further synchrony has been detected as the phase of a lower‐frequency (beta) oscillation is able to control the amplitude of a higher‐frequency (gamma) oscillation (Moran *et al*, 2011; De Hemptinne *et al*, 2015). This phase–amplitude coupling (De Hemptinne *et al*, 2015) has been observed in the motor cortex of PD patients with bradykinesia.

Thus, the current hypothesis of basal ganglia networks in PD is that the overall output to the thalamus and motor cortex is decreased and the circuit is caught in a hyper‐synchronized oscillatory state of entropy (Dorval *et al*, 2008; Anderson *et al*, 2015; Hoang *et al*, 2017). The efferent motor synapses of the STN during DBS are hypothesized to be depleted of their neurotransmitters and thereby filter low‐frequency oscillations (McIntyre & Anderson, 2016). Low‐frequency DBS (20 Hz) appears to increase the amount of synchronization, whereas high‐frequency DBS (> 70 Hz) suppresses such activity in the GPi (Brown *et al*, 2004), similar to the administration of levodopa (Dorval *et al*, 2008; Hoang *et al*, 2017).

The injection of postsynaptic noise and the decoupling of the STN neurons has been shown to reduce the power of beta oscillations, reduce phase/amplitude coupling, and decrease the entropy in the firing pattern of cortical neurons (Little & Brown, 2014; De Hemptinne *et al*, 2015; McIntyre & Anderson, 2016). This new firing pattern likely does not recreate the physiological firing pattern, but replaces the more deleterious intrinsic pattern of the corresponding circuitopathy. The overall network effect of STN‐DBS is an increased activity in the basal ganglia output from the thalamus to the motor cortex along with reduced synchrony in lower‐frequency oscillations.

## Neurochemical effects

**Figure 6:**
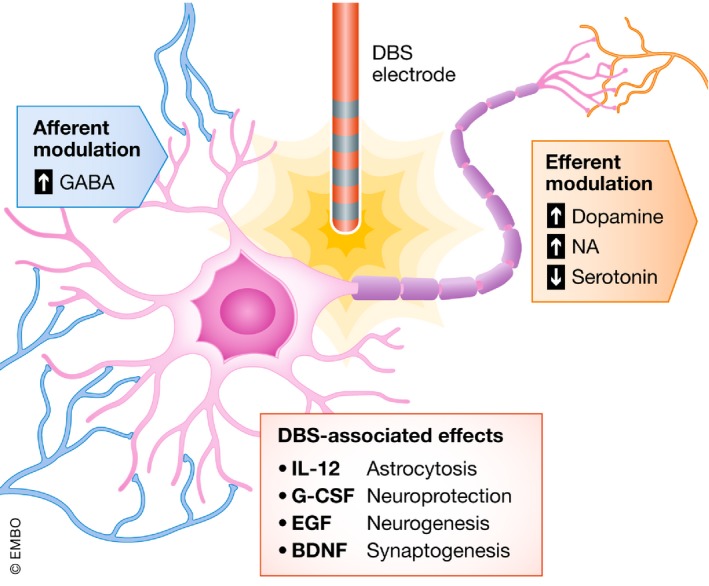
Effects of DBS on the molecular level Molecular effects of DBS: Inhibitory and excitatory neurotransmitters are up‐ or downregulated as afferent or efferent fibers are located in the volume of tissue activated. Growth factors and interleukins are known to be altered by DBS therapy in the context of Parkinson's disease.

DBS also seems to change the neurochemical milieu of neurons and neuronal tissue (Gondard *et al*, 2015; Udupa & Chen, 2015; Fischer *et al*, 2017; Torres‐Sanchez *et al*, 2017). Although the immediate effects of DBS are an altered firing pattern of neural circuits, these are often followed by changes in neurotransmitter dynamics and protein expression, which inevitably affect the behavior of the network in the longer term (McIntyre & Hahn, 2010; Fig 6).

### Dopamine

Dopamine is a well‐known therapeutic for treating movement disorders, such as PD, that are characterized by reduced dopamine production (Perlmutter & Mink, 2006). Animal models were thus used to quantify dopaminergic flux during and after DBS. In both anesthetized (Bruet *et al*, 2001; Meissner *et al*, 2001) and awake rats, STN‐DBS increases extracellular striatal dopamine in wild‐type and dopamine‐depleted animals (Meissner *et al*, 2002). Concomitant circling behavior was demonstrated during DBS, which suggests activation of gammaaminobutyric acid (GABA) receptors in the STN (Murer & Pazo, 1993). Complementary electrophysiologic studies in STN‐stimulated wild‐type rodents showed time‐locked increases in striatal dopamine efflux, as well as up to an 11‐fold chronic increase in dopamine after cessation of stimulation at 50 Hz and 200 μA (Lee *et al*, 2006).

In contrast to these animal studies, the majority of studies in PD patients undergoing STN‐DBS failed to show increased dopamine release, in spite of tremor amelioration during stimulation (Hilker *et al*, 2003; Strafella *et al*, 2003; Thobois *et al*, 2003). An isolated study in three PD patients, however, did suggest increased dopamine concentrations after stimulation (Nimura *et al*, 2005). The discrepancy in these experiments may result from differences in the precise subregion of the targeted STN (motor, affective, limbic part), which may affect the magnitude of dopamine release. Accordingly, in rhesus macaques, cyclic voltammetry demonstrated STN‐DBS‐evoked dopamine release in the caudate and putamen, with maximal concentration effects seen during stimulation of the posterior and lateral regions of the STN (Gale *et al*, 2013; Min *et al*, 2016). Notably, dopaminergic fibers are unmyelinated, which lowers their susceptibility to electrical stimulation compared to myelinated sites of DBS and may help explain the discrepancy in results (Kringelbach *et al*, 2007b). In summary, animal models have suggested DBS‐induced dopaminergic facilitation, though the findings have not been robustly substantiated in patients. This may be related to the fact that dopamine is greatly depleted in patients with advanced PD and thus unavailable for release in response to DBS.

### Noradrenaline

Noradrenaline (NA) might be involved in the mechanisms of action of DBS given the noradrenergic projections to the STN originating from the locus coeruleus. Depletion of NA and its metabolites within the limbic system has been implicated in both movement disorders and psychiatric pathology (Remy *et al*, 2005; Delaville *et al*, 2011). Specifically, NA is a key mediator of the cortico‐striato‐thalamo cortical pathway, components of which are targeted in DBS for obsessive–compulsive disorder (OCD) and major depressive disorder (MDD). Accordingly, bilateral nucleus accumbens (NAcc) DBS in rats yielded a sustained rise in dopamine and NA in the orbitofrontal cortex of up to 166% from baseline (Van Dijk *et al*, 2012). However, the same group found no increases in monoamine concentration near the stimulation site itself, suggesting that distal NA release dominates (Van Dijk *et al*, 2011). DBS of the infralimbic prefrontal cortex in a rodent depression model showed increased NA expression at the stimulation site, along with antidepressive effects (Jiménez‐Sánchez *et al*, 2016). More recently, DBS of the vmPFC (ventromedial prefrontal cortex: a rodent homolog to the human SCC) in a depression model helped to illuminate the electrophysiological mechanisms of DBS‐induced amine production (Torres‐Sanchez *et al*, 2018). Along with antidepressive effects, stimulation led to increased spontaneous firing of the locus coeruleus and increased expression of tyrosine hydroxylase, which catalyzes the biochemical synthesis of NA and other amines (Torres‐Sanchez *et al*, 2018).

### Serotonin

The serotonergic system is involved in prefrontal‐raphe limbic pathways, and its dysfunction has been implicated in mood disorders such as MDD. Recently, vmPFC‐DBS was found to increase serotonin levels by 33–55% in both wild‐type and serotonin transporter knockout mice while inducing antidepressive‐like behavior (Bregman *et al*, 2017). Similar experiments in rats showed up to 50% increase in serotonin in the hippocampus following vmPFC‐DBS (Boggio *et al*, 2005), as well as preferentially reversing anhedonia‐like behavior in animals with an intact serotonergic system (Hamani *et al*, 2012).

Recently, renewed appreciation of serotonin's role in the motor and non‐motor symptoms of PD has stimulated research into the serotonergic effects of DBS treatment for movement disorders. In a haloperidol‐induced tardive dyskinesia rat model, STN‐DBS resulted in reduced serotonin release from the dorsolateral striatum, correlating with reduced dyskinetic movements in the animals (Creed *et al*, 2012). However, the same experiment found that when serotonin reduction is pharmacologically blocked, STN‐DBS was still able to rescue the phenotype, suggesting that several parallel therapeutic mechanisms are involved (Creed *et al*, 2012). Other experiments have similarly found serotonin‐lowering effects of DBS in the hippocampus, prefrontal cortex (Navailles *et al*, 2010), and the striatum (Tan *et al*, 2013). A potential mechanism is electrical inhibition of dorsal raphe neurons that reside in the largest serotonergic nucleus of the brain (Temel *et al*, 2007). Though the STN provides few direct projections to the dorsal raphe proper, it does innervate the lateral habenula, which provides convergent input to the dorsal raphe nucleus, thereby suggesting a possible indirect serotonin‐modulating mechanism for STN‐DBS (Kalén *et al*, 1989; Temel *et al*, 2009).

### Gammaaminobutyric acid

Clinical studies have implicated activation of inhibitory GABAergic pathways as part of the therapeutic effects of electrical brain stimulation (Lozano & Eltahawy, 2004). For instance, intraoperative microstimulation of the GPi in PD patients was found to activate afferent striatal and GPe axons, resulting in GABA release and concurrent GPi inhibition (Dostrovsky *et al*, 2000). GPi, being the main output structure of the basal ganglia to the thalamus, therefore showed reduced GABAergic activity under STN‐DBS (Hashimoto *et al*, 2003; Miocinovic, 2006). Electrophysiological studies in primates revealed this inhibition to likely be mediated via GABA_A_ and GABA_B_ receptors (Chiken & Nambu, 2013). Similarly, in an STN‐DBS cohort of PD patients, both levodopa administration and electrical stimulation caused a decrease in GABA content in the motor thalamus, suggesting similar mechanism of motor symptom alleviation (Stefani *et al*, 2011). GABA levels were increased in the SNr during STN‐DBS, which showed normalized reduced neural activity (Tai, 2003) owing to decreased glutamatergic excitation. Since most DBS targets have both excitatory and inhibitory efferents and afferents, the overall effect on circuit behavior and phenotype is likely complex and is influenced by the relative distribution of these fibers (Lozano & Eltahawy, 2004; Montgomery & Gale, 2008).

## Neurochemical effects of electrode implantation

As previously mentioned, electrical effects of DBS underlie not only spatial but also temporal aspects. An animal study showed that implantation of a DBS electrode in the dorsal hippocampus has widespread and early effects on the brain even without electrical stimulation. The cellular effects were most pronounced after 2 weeks with microgliosis and astrocytosis; most of these cells were generated within 3 days after implantation (Song *et al*, 2013). The cytokine milieu showed expression of the pro‐inflammatory cytokine IL‐12, significant expression of granulocyte‐colony‐stimulating factor (G‐CSF) along with increased expression of epidermal growth factor (EGF) and brain‐derived neurotrophic factor (BDNF; Song *et al*, 2013). Immunohistochemical staining showed an increased pattern of doublecortin (DCX) positive cells—a marker for immature neurons—indicating neurogenesis in the hippocampal subgranular zone (Song *et al*, 2013). These early and fluid effects of electrode insertion have clinical implications as many centers do not start stimulation in the early phase after the surgery while the microenvironment around the electrode is still adapting to the effects of implantation.

In a prospective study, all patients showed a certain degree of peri‐electrode edema in MRI scans taken 3–20 days after the procedure and a rate of microhemorrhages of more than 30% (Borellini *et al*, 2018). This can be associated with temporary neurological deficits, states of confusion, or seizures (Deogaonkar *et al*, 2011; Arocho‐Quinones & Pahapill, 2016). Edema has not only been shown to be present at the tip of the electrode but also along the trajectory (Deogaonkar *et al*, 2011). Edema usually resolves after 4–6 weeks and, so far, there is no evidence that the presence or the size of edema correlates with the extent or duration of the insertional effect.

Microhemorrhages, edema, and other reactions around the electrode can change the tissue impedance, especially within the first days after surgery, but also over longer terms (Hartmann *et al*, 2015). When utilizing a voltage‐controlled DBS system, impedance changes theoretically cause varying currents and therefore VTAs, which can lead to under‐, or more common, overstimulation, and side effects. Current‐controlled DBS systems that adapt the voltage to the impedances may therefore be more suitable for earlier programming (Picillo *et al*, 2016). However, to date no evidence‐based guidelines for when to start stimulation exist. Some centers prefer early programming after surgery while others wait 4 weeks or more (depending on the microlesional effect) until they begin stimulation (Kupsch *et al*, 2011; Picillo *et al*, 2016).

To determine long‐term effects on the tissue surrounding the electrode, cadaver studies of PD patients with STN‐DBS showed an up to 1‐mm‐thick capsule around the electrode with a characteristic three‐layer structure. The inner layer was a thin 25‐μm membrane of fibrous tissue. The next layer was a 500‐μm‐thick rim of fibrillary gliosis followed by an up to 1,000‐μm‐thick layer consisting of reactive astrocytes (Reddy & Lozano, 2017). Signs of a microhemorrhage in the form of hemosiderin as a part of the insertional or microlesional effect were also present as well as signs of immune response in the form of t‐lymphocytes and multinucleated giant cells (Reddy & Lozano, 2017). Whether this is attributed to direct effects of the stimulation or a foreign‐body reaction is unclear.

Vascular changes have been observed too. Endothelial wall thickness was found to be reduced in PD patients compared to non‐PD subjects; however, patients with STN‐DBS showed increased thickness even compared to non‐PD subjects (Pienaar *et al*, 2015). Although wall thickness increased with STN‐DBS, tight junction proteins were found to be reduced compared to non‐DBS PD patients (Pienaar *et al*, 2015). This, together with the finding of an increased capillary density, and increased expression of vascular endothelial growth factor (VEGF) and glial‐derived neurotrophic factor (GDNF), led to the hypothesis that STN‐DBS changes the microvasculature and causes sprouting of new more stables vessels compared to PD patients without DBS therapy (Pienaar *et al*, 2015).

## Longer‐term effects

Changes in neurotransmitter release, expression, and receptor dynamics after DBS can have network and biochemical effects that transcend the time and spatial zone of stimulation (Deniau *et al*, 2010; McIntyre & Hahn, 2010). This finding has spurred pre‐ and clinical research into novel applications of DBS, such as treatment of dementia‐related disorders (Laxton *et al*, 2010; Hescham *et al*, 2013; Sankar *et al*, 2015), stroke (Elias *et al*, 2018), pain syndromes (Owen *et al*, 2006; Russo *et al*, 2018), and psychiatric disease (Holtzheimer & Mayberg, 2011).

### Synaptic and neural plasticity

Recent advances in functional and structural neuroimaging have enabled mapping the spatial and temporal effects of DBS across the whole brain. In the first demonstration of magnetoencephalography (MEG) to investigate whole‐brain changes, DBS of the right periventricular gray/periaqueductal gray (PVG/PAG) for treating phantom limb pain was shown to increase activity in a network, including subgenual cingulate and mid‐anterior orbitofrontal cortex, associated with pain relief (Kringelbach *et al*, 2007a). More recently, MEG was used to show that DBS of the anterior cingulate cortex (ACC) for chronic pain induced long‐term functional changes in a pain‐associated network for up to 1 year after surgery (Mohseni *et al*, 2012). Non‐invasive characterization of functional connectivity during STN‐DBS for PD is of particular interest, as it allows to study the higher‐order circuit dynamics and mechanism of action. Using functional MRI (fMRI), STN‐DBS was found to desensitize the STN of its afferent, while simultaneously strengthening corticostriatal, thalamocortical, and direct pathways (Kahan *et al*, 2014).

Although the neurochemical correlates are not precisely established in humans, preclinical studies in a 6‐hydroxydopamine‐lesioned PD rat model found that STN‐DBS for 1 h increased relative synaptic connectivity among corticostriatal glutamatergic terminals, thereby potentially contributing to the therapeutic benefit by partially rescuing striatal glutamatergic pathways (Walker *et al*, 2012). STN‐DBS in hemi‐parkinsonian rats showed duration‐dependent metabolic effects for up to 1 week, such as counteracting lesion‐induced glutamate/glutamine/GABA increases in the striatum, and normalizing GABA in the SNr, concomitant with a therapeutic effect on motor phenotype (Melon *et al*, 2015). Transcranial magnetic stimulation (TMS) is another useful non‐invasive method to study long‐ and short‐term plasticity during DBS, via paired‐pulse stimulation and motor evoked potential (MEP) recording. Short‐interval intracortical inhibition associated with motor improvement has been observed in STN and GPi DBS for PD, suggesting a restoration of the thalamocortical pathway during stimulation (Pierantozzi *et al*, 2002; Kim *et al*, 2015). Other network effects include enhancement of cortical reactivity, suggesting possible antidromic excitation of fibers connecting STN and motor cortex (Hanajima *et al*, 2004; Casula *et al*, 2017).

Structural and functional connectivity studies have begun to illuminate the importance of network effects beyond the target nucleus and basal ganglia circuit. A recent study investigated connectivity as a predictor of DBS outcome by using retrospective clinical data from PD patients who underwent STN‐DBS at two centers. The authors used a publicly available connectome database acquired via diffusion tractography and resting‐state functional connectivity to generate a connectivity profile for each patient (Horn *et al*, 2017). They found that certain connectivity profiles reliably predicted clinical outcome independent of electrode location, suggesting that network‐wide phenomena might be as important as local molecular effects (Horn *et al*, 2017). Specifically, connectivity from the DBS target to the supplementary motor area and primary motor cortex was highly predictive for clinical outcome, corroborating evidence in TMS studies targeting these cortical regions (Shirota *et al*, 2013; Brys *et al*, 2016; Horn *et al*, 2017). Further prospective studies are needed that examine the relationship between electrode location, anatomic and functional connectivity, and disease‐relevant clinical outcomes.

### Epigenetic effects

DNA‐methylation status in brain and blood samples from PD patients show high levels of congruence (Consales *et al*, 2018). Thus, a transcription fingerprint of leukocytes from PD patients was proposed, as these have been shown to respond to different brain pathologies such as stroke (Soreq *et al*, 2013). A distinct pattern of microRNAs between healthy subjects, PD patients under medical treatment, and PD patients with DBS was observed (Soreq *et al*, 2013). Changes in the methylation pattern were even observed after 1 h without stimulation, suggesting rapid miRNA responses (Soreq *et al*, 2013) related to inflammatory control and protection against DNA damage. The authors suggest that leukocyte methylation patterns could be used as biomarkers for effective DBS treatment of PD. The practical clinical implications might be individualized and adjustable DBS therapy based on peripheral epigenetic fingerprints, much in the same way that serial neurological assessment is used to titrate DBS contacts and voltage (Mohammadi & Mehdizadeh, 2016; Consales *et al*, 2018). Nevertheless, clinical application is likely years away, as further validation of epigenetic biomarkers is needed to determine the sensitivity, specificity, and clinical correlates of such peripheral measurements.

### Neurogenesis, synaptogenesis, and neuroprotection

Growing preclinical evidence suggests that, in addition to alleviating symptoms, STN‐DBS has disease‐modifying effects such as slowing nigral degeneration and neuroprotection. Rodent models have shown that chronic STN‐DBS can lead to reduced loss of tyrosine‐hydroxylase‐positive (TH^+^) nigral neurons compared to sham stimulation (Temel *et al*, 2006; Spieles‐Engemann *et al*, 2010; Fischer *et al*, 2017; Musacchio *et al*, 2017). Tyrosine hydroxylase is essential for metabolizing l‐tyrosine to levodopa. In line with these observations, Wallace *et al* tested STN‐DBS on non‐human primates with acute bilateral MPTP‐mediated (methyl‐phenyl‐tetrahydropyridine) SNc TH^+^ lesions; the animals received either unilateral STN lesions or DBS stimulation for a week. The authors found 20–24% more TH^+^ DA cells in the SNc ipsilateral to stimulation or lesion, suggesting a protective effect of DBS (Wallace *et al*, 2007).

Recently, neurotrophic mechanisms of STN‐DBS neuroprotection have also been demonstrated in preclinical models of PD (Spieles‐Engemann *et al*, 2010; Fischer *et al*, 2017; Fischer & Sortwell, 2018). Specifically, *in vivo* studies showed that STN‐DBS promotes expression of BDNF in the basal ganglia, which in turn binds to the tropomyosin‐related kinase type 2 (trkB) receptor. As the chief BDNF and neurotropin‐3 receptor in the basal ganglia, trkB has intermediate and long‐term downstream effects including promoting dopaminergic neuron survival (Baydyuk *et al*, 2011), regulating synaptic plasticity (Leal *et al*, 2014), and maintenance of the nigrostriatal pathway as a whole (Baydyuk *et al*, 2011; Fischer *et al*, 2017). Increased BDNF was seen with high‐frequency electrical stimulation of cultured dopaminergic neurons (Hartmann *et al*, 2001), as well as STN‐DBS of PD rodent models (Fischer *et al*, 2017), with long‐lasting neuroprotective benefits in the latter even after discontinuation of stimulation. Similarly, studies of STN‐DBS in non‐human primate models of PD have shown potential neuroprotective effects via inhibition of excitotoxic cell death (Tsukahara *et al*, 1995). Other proposed neuroprotective mechanisms include the following: promoting synaptogenesis via sustained trkB signalizing (Guo *et al*, 2018) and facilitating striatal dopaminergic neurotransmission via maintenance of dendritic spine density (Rauskolb *et al*, 2010). Another theory, called electrotaxis*,* describes how the speed and directionality of precursor cell movement could be facilitated by the electric field generated from the DBS electrode (Jahanshahi *et al*, 2014).

Although post‐mortem studies have found increased neural precursors in the subventricular zones of patients with chronic PD (Vedam‐Mai *et al*, 2014), human trials to date have failed to show a direct disease‐modifying effect of STN‐DBS. Given that striatal dopaminergic dysfunction and neural loss are already well‐established at the time of diagnosis, and that DBS is typically considered after 4 years of symptoms, it is perhaps unsurprising that surgical intervention has failed to produce any detectable disease modification in clinical trials. Moreover, PD patients enrolled in DBS trials are typically at that late stages of the disease, where the purpose of electrical stimulation is largely symptomatic. The design of future clinical trials should include more sensitive biomarkers and functional assessments of PD neuropathogenesis, and intervention at earlier stages where striatal neural salvage might still be possible (Fischer & Sortwell, 2018).

When applied to structures within the circuit of Papez, DBS has also been found to exert beneficial effects in neuropathological hallmarks, molecular expression, and behavior. Bilateral fornix DBS in the rat for 1‐h induced expression of cFos, an immediate‐early marker of neural activation, in the hippocampus. BDNF and VEGF were also significantly increased 2.5 h after stimulation, suggesting that neurotrophic and proliferating factors are associated with electrical stimulation (Gondard *et al*, 2015). Whether one would expect a constant increase in biochemical biomarkers before reaching a stable plateau or whether these markers show natural fluctuation under stimulation is so far not known. This might have future implications for cycled stimulation paradigms.

A similar influence on BDNF has been observed in a case study of chronic bilateral DBS of the lateral habenula for treatment‐resistant depression: BDNF levels correlated in a reverse U‐shape with levels of depression scores (Hoyer & Sartorius, 2012). Chronic fornix DBS was performed in a transgenic Alzheimer's disease (AD) mouse model and showed rescue of memory impairment as well as amyloid‐beta plaque clearance in the subiculum (Mann *et al*, 2017). The entorhinal cortex is another DBS target to rescue both early memory deficits and early plaque burden in a different AD mouse model, potentially mediated by direct projections from the entorhinal cortex to the hippocampus (Xia *et al*, 2017). Based on preliminary phase 1 studies in patients with improved cognition, reduction in cerebral atrophy, and cortical glucose metabolism positron emission tomography (PET) studies, an ongoing multi‐national clinical trial of fornix DBS for AD is currently underway (Laxton *et al*, 2010; Sankar *et al*, 2014, 2015; Mirzadeh *et al*, 2016). Both fornix and entorhinal DBS have been shown to increase hippocampal neurogenesis in normal rodents (Stone *et al*, 2011) and disease models of Rett syndrome and AD.

## Clinical effects of DBS

The cumulative effects of DBS therapy on the cellular, molecular, or electrical microlevel add up to clinically observable effects on the macrolevel for a variety of indications.

During the early years, DBS was applied to only the most severe cases of PD with a mean history of 11–13 years after diagnosis. As the therapy showed positive results, it was speculated if an earlier intervention might be beneficial. In a multicenter trial, 251 patients with only 7.5 years of history of PD and early motor complications were randomized to receive either the best medical therapy or the DBS. After 2 years, quality of life improved significantly in the DBS group, while it slightly decreased for patients with best medical treatment. Neurostimulation was also superior in regard to motor disability, activities of daily living, and levodopa‐induced dyskinesias (Schuepbach *et al*, 2013). The levodopa dose was reduced by 39% in the DBS group while it was increased by 21% in the group with best medical treatment. Hypo‐ and hyperdopaminergic behavioral symptoms (like apathy or mania) were significantly reduced in the neurostimulation vs. the medical treatment group (Lhommée *et al*, 2018).

In the case of essential tremor, DBS has shown to lead to 60–75% long‐term tremor control in more than 1,000 patients since 1998 (Dallapiazza *et al*, 2018). As these patients are for the most part compromised in daily activities (buttoning a shirt, holding a cup, using fork and knife, writing), it is no surprise that overall quality of life improved from 57.9 to 82% with DBS therapy in these patients (Dallapiazza *et al*, 2018).

Another standard indication for DBS is the treatment of dystonia. A meta‐analysis of 523 patients with inherited or idiopathic dystonia showed a 65.2% reduction in dystonia motor rating scales after a mean 32.5 months of follow up. Disability scores were reduced with DBS therapy by 58.6% (Moro *et al*, 2017). As generalized dystonia especially affects pediatric patients, DBS represents an invasive but very effective therapy. A recent meta‐analysis of 321 pediatric patients treated with DBS found that 86.3% showed a general and 66.1% a clinically significant improvement in dystonia scores (Elkaim *et al*, 2018).

In a multicenter study of therapy‐refractory partial or secondarily generalized epilepsy, 110 patients received DBS in the anterior nucleus of the thalamus (ANT) and were randomized to active or inactive stimulation. After a 3‐month blinded phase, the group with active DBS had a significantly higher reduction in seizure frequency (−40.4%) than the group without stimulation (−14.5%). After 2 years of active stimulation, the seizure frequency was reduced by 56% compared to baseline (Fisher *et al*, 2010). DBS therapy showed improvements of 47.7% in scores for 108 patients with obsessive–compulsive disorder and an overall responder rate (with a clinically meaningful reduction of 35% or more in the OCD score) of 58.2% (van Westen *et al*, 2015).

In summary, DBS positively affects the main symptoms of the corresponding disease and thereby reduces the amount of medication needed while increasing the quality of life for a large proportion of treated patients.

## Current and future developments

**Figure 7:**
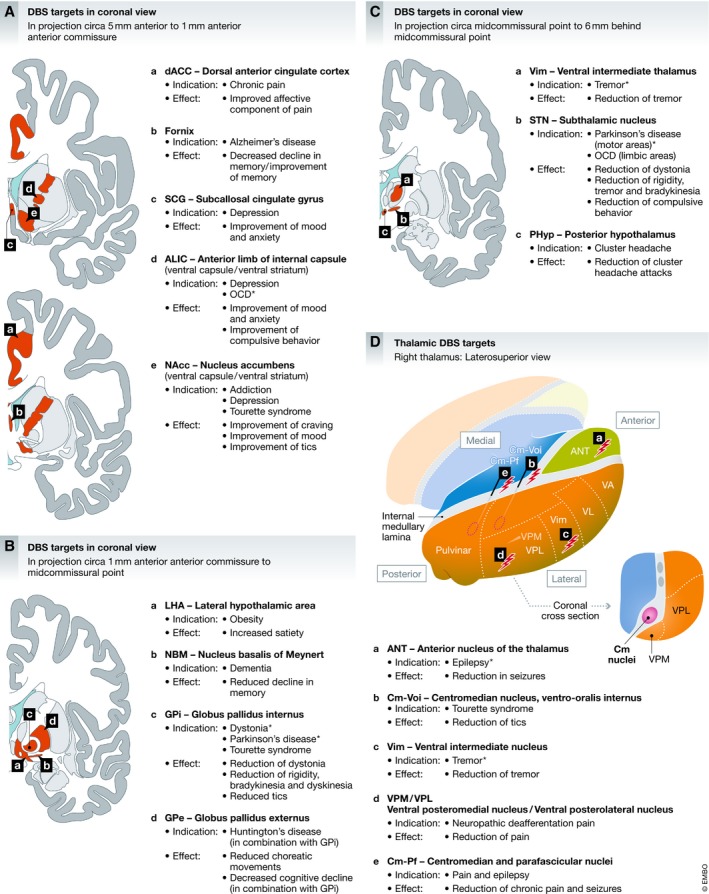
Overview of selected targets for DBS Location of DBS targets for established and investigational indications: (A) DBS targets in coronal projection view (rostral to anterior commissure), (B) DBS targets in coronal projection view (anterior commissure to midcommissural point), (C) midcommissural point to posterior commissure), and (D) overview of thalamic DBS targets.

A more complete understanding of the different mechanisms through which DBS exerts its action has opened up the field for new clinical indications, new stimulation targets, and new ways to deliver stimulation to the brain (Fig 7A–D). Various psychiatric circuitopathies have been explored with DBS therapy. Multiple targets such as the subgenual cingulate cortex (Brodmann Area 25, CG25; Holtzheimer *et al*, 2017), the NAcc (Bewernick *et al*, 2010; Fig 7A), the anterior limb of the internal capsule (ALIC; Bergfeld *et al*, 2016; Fig 7A), the medial forebrain bundle (Schlaepfer *et al*, 2013), and the lateral habenula (Sartorius *et al*, 2010) were investigated for treatment of MDD. DBS for treating OCD has been performed in the anterior limb of the internal capsule (Goodman *et al*, 2010), the bed nucleus of the stria terminalis (Nuttin *et al*, 2013), and the STN (Chabardès *et al*, 2013; Fig 7C) and the NAcc (Huff *et al*, 2010; Fig 7A). The NAcc has also been used to treat different forms of addiction (Müller *et al*, 2016). Tourette's syndrome has been treated with DBS in the NAcc (Kuhn *et al*, 2007), globus pallidus internus (Akbarian‐Tefaghi *et al*, 2017; Fig 7B), and the ventral anterior thalamus (Voa; Huys *et al*, 2016; Fig 7D). Eating disorders such as anorexia nervosa (CG25; Lipsman *et al*, 2013) or morbid obesity (with NAcc and lateral hypothalamic DBS; Whiting *et al*, 2013; Tronnier *et al*, 2018) have also been investigated as potential indications for DBS therapy. DBS of the fornix (Lozano *et al*, 2016; Fig 7A) and the nucleus basalis of Meynert (Hardenacke *et al*, 2013) is being explored as a treatment for Alzheimer's. Combined stimulation of GPi and GPe (Fig 7B) is currently being explored for treating Huntington's disease to reduce the amount of choreatic movements and cognitive decline (Wojtecki *et al*, 2016).

As the traditional indication for DBS therapy, movement disorders continually see new developments to improve effectiveness or efficiency. Adaptive closed‐loop DBS systems can detect disease‐ and state‐specific biomarkers and adapt stimulation parameters delivered to the brain. This can include the power of beta oscillations in PD (Little *et al*, 2016) or accelerometer‐detected amplitude in tremor (Herron *et al*, 2017). Other forms of stimulation rhythms that differ from current monophasic tonic stimulation are being explored, for example, with stimulation patterns similar to the synaptic noise generated by DBS (Leong *et al*, 2018).

## Conclusion

Although the effects of DBS may often mimic a temporary lesion, the underlying mechanisms of action by far surpass simple inactivation of neurons. DBS has complex electrical effects on single neurons and neuronal networks, changes neurotransmitter concentrations and dynamics, and shapes the microenvironment including astrocytes, microglia, and endothelial cells. DBS also influences neuroplasticity and possibly induces neuroprotection and neurogenesis. With stereotactic implantation of electrodes with high precision and the possibility to modulate and titrate therapeutic effects, DBS is rapidly becoming a powerful tool to study brain function and physiology and an effective treatment for many different diseases.

## Author contributions

MJ: manuscript draft, literature research, and figures; AF: manuscript draft and literature research; AML: correction; and KLK: conception of article, manuscript draft, and correction.

## Conflict of interest

The authors declare that they have no conflict of interest.

**Pending issues**

(i) *In vitro* electrophysiology studies to refine electrical and neurochemical mechanisms
(ii) Effects of DBS on vasculature, blood–brain barrier, and glia (non‐neural tissue)
(iii) Identifying optimal DBS targets for each indication
(iv) Refining parameters for DBS such as frequency, pulse width, and duration
(v) Prospective, randomized, and blinded trials on outcomes of novel indications for DBS
(vi) Closed‐loop neuromodulation to individualize DBS.

## References

[emmm201809575-bib-0001] Agnesi F , Connolly AT , Baker KB , Vitek JL , Johnson MD (2013) Deep brain stimulation imposes complex informational lesions. PLoS One 8: e74462 23991221 10.1371/journal.pone.0074462 PMC3753277

[emmm201809575-bib-0002] Akbarian‐Tefaghi L , Akram H , Johansson J , Zrinzo L , Kefalopoulou Z , Limousin P , Joyce E , Hariz M , Wårdell K , Foltynie T (2017) Refining the deep brain stimulation target within the limbic globus pallidus internus for Tourette syndrome. Stereotact Funct Neurosurg 95: 251–258 28787721 10.1159/000478273

[emmm201809575-bib-0003] Ammari R , Bioulac B , Garcia L , Hammond C (2011) The subthalamic nucleus becomes a generator of bursts in the dopamine‐depleted state. Its high frequency stimulation dramatically weakens transmission to the globus pallidus. Front Syst Neurosci 5: 43 21716635 10.3389/fnsys.2011.00043 PMC3115486

[emmm201809575-bib-0004] Anderson T , Hu B , Pittman Q , Kiss ZHT (2004) Mechanisms of deep brain stimulation: an intracellular study in rat thalamus. J Physiol 559: 301–313 15218068 10.1113/jphysiol.2004.064998 PMC1665080

[emmm201809575-bib-0005] Anderson CJ , Sheppard DT , Huynh R , Anderson DN , Polar CA , Dorval AD (2015) Subthalamic deep brain stimulation reduces pathological information transmission to the thalamus in a rat model of Parkinsonism. Front Neural Circuits 9: 31 26217192 10.3389/fncir.2015.00031 PMC4491629

[emmm201809575-bib-0006] Arocho‐Quinones EV , Pahapill PA (2016) Non‐infectious peri‐electrode edema and contrast enhancement following deep brain stimulation surgery. Neuromodulation 19: 872–876 27098925 10.1111/ner.12432

[emmm201809575-bib-0007] Baker KB , Montgomery EB , Rezai AR , Burgess R , Lüders HO (2002) Subthalamic nucleus deep brain stimulus evoked potentials: physiological and therapeutic implications. Mov Disord 17: 969–983 12360546 10.1002/mds.10206

[emmm201809575-bib-0008] Barat E , Boisseau S , Bouyssières C , Appaix F , Savasta M , Albrieux M (2012) Subthalamic nucleus electrical stimulation modulates calcium activity of nigral astrocytes. PLoS One 7: e41793 22848608 10.1371/journal.pone.0041793 PMC3407058

[emmm201809575-bib-0009] Başar E , Başar‐Eroǧlu C , Güntekin B , Yener GG (2013) Brain's alpha, beta, gamma, delta, and theta oscillations in neuropsychiatric diseases: proposal for biomarker strategies. Suppl Clin Neurophysiol 62: 19–54 24053030 10.1016/b978-0-7020-5307-8.00002-8

[emmm201809575-bib-0010] Baydyuk M , Nguyen MT , Xu B (2011) Chronic deprivation of TrkB signaling leads to selective late‐onset nigrostriatal dopaminergic degeneration. Exp Neurol 228: 118–125 21192928 10.1016/j.expneurol.2010.12.018 PMC3041148

[emmm201809575-bib-0011] Benabid AL , Pollak P , Gao D , Hoffmann D , Limousin P , Gay E , Payen I , Benazzouz A (1996) Chronic electrical stimulation of the ventralis intermedius nucleus of the thalamus as a treatment of movement disorders. J Neurosurg 84: 203–214 8592222 10.3171/jns.1996.84.2.0203

[emmm201809575-bib-0012] Benazzouz A , Hallett M (2000) Mechanism of action of deep brain stimulation. Neurology 55: S13–S16 11188968

[emmm201809575-bib-0013] Bergfeld IO , Mantione M , Hoogendoorn MLC , Ruhé HG , Notten P , van Laarhoven J , Visser I , Figee M , de Kwaasteniet BP , Horst F *et al* (2016) Deep brain stimulation of the ventral anterior limb of the internal capsule for treatment‐resistant depression. JAMA Psychiatry 73: 456–464 27049915 10.1001/jamapsychiatry.2016.0152

[emmm201809575-bib-0014] Beurrier C , Bioulac B , Audin J , Hammond C (2001) High‐frequency stimulation produces a transient blockade of voltage‐gated currents in subthalamic neurons. J Neurophysiol 85: 1351–1356 11287459 10.1152/jn.2001.85.4.1351

[emmm201809575-bib-0015] Bewernick BH , Hurlemann R , Matusch A , Kayser S , Grubert C , Hadrysiewicz B , Axmacher N , Lemke M , Cooper‐Mahkorn D , Cohen MX *et al* (2010) Nucleus accumbens deep brain stimulation decreases ratings of depression and anxiety in treatment‐resistant depression. Biol Psychiatry 67: 110–116 19914605 10.1016/j.biopsych.2009.09.013

[emmm201809575-bib-0016] Boggio PS , Fregni F , Bermpohl F , Mansur CG , Rosa M , Rumi DO , Barbosa ER , Rosa MO , Pascual‐Leone A , Rigonatti SP *et al* (2005) Effect of repetitive TMS and fluoxetine on cognitive function in patients with Parkinson's disease and concurrent depression. Mov Disord 20: 1178–1184 15895421 10.1002/mds.20508

[emmm201809575-bib-0017] Borellini L , Ardolino G , Carrabba G , Locatelli M , Rampini P , Sbaraini S , Scola E , Avignone S , Triulzi F , Barbieri S *et al* (2018) Peri‐lead edema after deep brain stimulation surgery for Parkinson's disease: a prospective magnetic resonance imaging study. Eur J Neurol 26: 533–539 30358915 10.1111/ene.13852

[emmm201809575-bib-0018] Bregman T , Nona C , Volle J , Diwan M , Raymond R , Fletcher PJ , Nobrega JN , Hamani C (2017) Deep brain stimulation induces antidepressant‐like effects in serotonin transporter knockout mice. Brain Stimul 11: 423–425 29174865 10.1016/j.brs.2017.11.008

[emmm201809575-bib-0019] Brown RG , Limousin Dowsey P , Brown P , Jahanshahi M , Pollak P , Benabid AL , Rodriguez‐Oroz MC , Obeso J , Rothwell JC (1999) Impact of deep brain stimulation on upper limb akinesia in Parkinson's disease. Ann Neurol 45: 473–488 10211472 10.1002/1531-8249(199904)45:4<473::aid-ana9>3.0.co;2-v

[emmm201809575-bib-0020] Brown P , Mazzone P , Oliviero A , Altibrandi MG , Pilato F , Tonali PA , Di Lazzaro V (2004) Effects of stimulation of the subthalamic area on oscillatory pallidal activity in Parkinson's disease. Exp Neurol 188: 480–490 15246847 10.1016/j.expneurol.2004.05.009

[emmm201809575-bib-0021] Brown P , Williams D (2005) Basal ganglia local field potential activity: character and functional significance in the human. Clin Neurophysiol 116: 2510–2519 16029963 10.1016/j.clinph.2005.05.009

[emmm201809575-bib-0022] Bruet N , Windels F , Bertrand A , Feuerstein C , Poupard A , Savasta M (2001) High frequency stimulation of the subthalamic nucleus increases the extracellular contents of striatal dopamine in normal and partially dopaminergic denervated rats. J Neuropathol Exp Neurol 60: 15–24 11202172 10.1093/jnen/60.1.15

[emmm201809575-bib-0023] Brys M , Fox MD , Agarwal S , Biagioni M , Dacpano G , Kumar P , Pirraglia E , Chen R , Wu A , Fernandez H *et al* (2016) Multifocal repetitive TMS for motor and mood symptoms of Parkinson disease. Neurology 87: 1907–1915 27708129 10.1212/WNL.0000000000003279 PMC5100715

[emmm201809575-bib-0024] Carron R , Chaillet A , Filipchuk A , Pasillas‐Lépine W , Hammond C (2013) Closing the loop of deep brain stimulation. Front Syst Neurosci 7: 112 24391555 10.3389/fnsys.2013.00112 PMC3868949

[emmm201809575-bib-0025] Casula EP , Stampanoni Bassi M , Pellicciari MC , Ponzo V , Veniero D , Peppe A , Brusa L , Stanzione P , Caltagirone C , Stefani A *et al* (2017) Subthalamic stimulation and levodopa modulate cortical reactivity in Parkinson's patients. Parkinsonism Relat Disord 34: 31–37 27771287 10.1016/j.parkreldis.2016.10.009

[emmm201809575-bib-0026] Chabardès S , Polosan M , Krack P , Bastin J , Krainik A , David O , Bougerol T , Benabid AL (2013) Deep brain stimulation for obsessive‐compulsive disorder: subthalamic nucleus target. World Neurosurg 80: S31.e1–8 10.1016/j.wneu.2012.03.010 22469523

[emmm201809575-bib-0027] Chiken S , Nambu A (2013) High‐frequency pallidal stimulation disrupts information flow through the pallidum by GABAergic inhibition. J Neurosci 33: 2268–2280 23392658 10.1523/JNEUROSCI.4144-11.2013 PMC6619164

[emmm201809575-bib-0028] Consales C , Merla C , Marino C , Benassi B (2018) The epigenetic component of the brain response to electromagnetic stimulation in Parkinson's disease patients: a literature overview. Bioelectromagnetics 39: 3–14 28990199 10.1002/bem.22083

[emmm201809575-bib-0029] Creed MC , Hamani C , Bridgman A , Fletcher PJ , Nobrega JN (2012) Contribution of decreased serotonin release to the antidyskinetic effects of deep brain stimulation in a rodent model of tardive dyskinesia: comparison of the subthalamic and entopeduncular nuclei. J Neurosci 32: 9574–9581 22787043 10.1523/JNEUROSCI.1196-12.2012 PMC6622267

[emmm201809575-bib-0030] Dallapiazza RF , Lee DJ , De Vloo P , Fomenko A , Hamani C , Hodaie M , Kalia SK , Fasano A , Lozano AM (2018) Outcomes from stereotactic surgery for essential tremor. J Neurol Neurosurg Psychiatry. 10.1136/jnnp-2018-318240 PMC6581115 30337440

[emmm201809575-bib-0031] De Hemptinne C , Swann NC , Ostrem JL , Ryapolova‐Webb ES , San Luciano M , Galifianakis NB , Starr PA (2015) Therapeutic deep brain stimulation reduces cortical phase‐amplitude coupling in Parkinson's disease. Nat Neurosci 18: 779–786 25867121 10.1038/nn.3997 PMC4414895

[emmm201809575-bib-0032] Degos B (2005) Neuroleptic‐induced catalepsy: electrophysiological mechanisms of functional recovery induced by high‐frequency stimulation of the subthalamic nucleus. J Neurosci 25: 7687–7696 16107655 10.1523/JNEUROSCI.1056-05.2005 PMC6725399

[emmm201809575-bib-0033] Dejean C , Hyland B , Arbuthnott G (2009) Cortical effects of subthalamic stimulation correlate with behavioral recovery from dopamine antagonist induced akinesia. Cereb Cortex 19: 1055–1063 18787234 10.1093/cercor/bhn149

[emmm201809575-bib-0034] Delaville C , De Deurwaerdère P , Benazzouz A (2011) Noradrenaline and Parkinson's disease. Front Syst Neurosci 5: 31 21647359 10.3389/fnsys.2011.00031 PMC3103977

[emmm201809575-bib-0035] Deniau JM , Degos B , Bosch C , Maurice N (2010) Deep brain stimulation mechanisms: beyond the concept of local functional inhibition. Eur J Neurosci 32: 1080–1091 21039947 10.1111/j.1460-9568.2010.07413.x

[emmm201809575-bib-0036] Deogaonkar M , Nazzaro JM , Machado A , Rezai A (2011) Transient, symptomatic, post‐operative, non‐infectious hypodensity around the deep brain stimulation (DBS) electrode. J Clin Neurosci 18: 910–915 21571534 10.1016/j.jocn.2010.11.020

[emmm201809575-bib-0037] Dorval AD , Russo GS , Hashimoto T , Xu W , Grill WM , Vitek JL (2008) Deep brain stimulation reduces neuronal entropy in the MPTP‐primate model of Parkinson's disease. J Neurophysiol 100: 2807–2818 18784271 10.1152/jn.90763.2008 PMC2585386

[emmm201809575-bib-0038] Dostrovsky JO , Levy R , Wu JP , Hutchison WD , Tasker RR , Lozano AM (2000) Microstimulation‐induced inhibition of neuronal firing in human globus pallidus. J Neurophysiol 84: 570–574 10899228 10.1152/jn.2000.84.1.570

[emmm201809575-bib-0039] Dostrovsky JO , Lozano AM (2002) Mechanisms of deep brain stimulation. Mov Disord 17: S63–S68 10.1002/mds.10143 11948756

[emmm201809575-bib-0040] Elias GJB , Namasivayam AA , Lozano AM (2018) Deep brain stimulation for stroke: current uses and future directions. Brain Stimul 11: 3–28 29089234 10.1016/j.brs.2017.10.005

[emmm201809575-bib-0041] Elkaim LM , Alotaibi NM , Sigal A , Alotaibi HM , Lipsman N , Kalia SK , Fehlings DL , Lozano AM , Ibrahim GM (2018) Deep brain stimulation for pediatric dystonia: a meta‐analysis with individual participant data. Dev Med Child Neurol 61: 49–56 30320439 10.1111/dmcn.14063

[emmm201809575-bib-0042] Fenoy AJ , Goetz L , Chabardès S , Xia Y (2014) Deep brain stimulation: are astrocytes a key driver behind the scene?. CNS Neurosci Ther 20: 191–201 24456263 10.1111/cns.12223 PMC3969941

[emmm201809575-bib-0043] Fischer DL , Kemp CJ , Cole‐Strauss A , Polinski NK , Paumier KL , Lipton JW , Steece‐Collier K , Collier TJ , Buhlinger DJ , Sortwell CE (2017) Subthalamic nucleus deep brain stimulation employs trkB signaling for neuroprotection and functional restoration. J Neurosci 37: 6786–6796 28607168 10.1523/JNEUROSCI.2060-16.2017 PMC5508259

[emmm201809575-bib-0044] Fischer DL , Sortwell CE (2018) BDNF provides many routes toward STN DBS‐mediated disease modification. Mov Disord 34: 22–34 30440081 10.1002/mds.27535 PMC6587505

[emmm201809575-bib-0045] Fisher R , Salanova V , Witt T , Worth R , Henry T , Gross R , Oommen K , Osorio I , Nazzaro J , Labar D *et al* (2010) Electrical stimulation of the anterior nucleus of thalamus for treatment of refractory epilepsy. Epilepsia 51: 899–908 20331461 10.1111/j.1528-1167.2010.02536.x

[emmm201809575-bib-0046] Florence G , Dahlem MA , Almeida ACG , Bassani JWM , Kurths J (2009) The role of extracellular potassium dynamics in the different stages of ictal bursting and spreading depression: a computational study. J Theor Biol 22: 332–345 10.1016/j.jtbi.2009.01.032 19490858

[emmm201809575-bib-0047] Gale JT , Lee KH , Amirnovin R , Roberts DW , Williams ZM , Blaha CD , Eskandar EN (2013) Electrical stimulation‐evoked dopamine release in the primate striatum. Stereotact Funct Neurosurg 91: 355–363 24107983 10.1159/000351523

[emmm201809575-bib-0048] Gondard E , Chau HN , Mann A , Tierney TS , Hamani C , Kalia SK , Lozano AM (2015) Rapid modulation of protein expression in the rat hippocampus following deep brain stimulation of the fornix. Brain Stimul 8: 1058–1064 26321354 10.1016/j.brs.2015.07.044

[emmm201809575-bib-0049] Goodman WK , Foote KD , Greenberg BD , Ricciuti N , Bauer R , Ward H , Shapira NA , Wu SS , Hill CL , Rasmussen SA (2010) Deep brain stimulation for intractable obsessive compulsive disorder: pilot study using a blinded, staggered‐onset design. Biol Psychiatry 67: 535–542 20116047 10.1016/j.biopsych.2009.11.028 PMC5796545

[emmm201809575-bib-0050] Gradinaru V , Mogri M , Thompson KR , Henderson JM , Deisseroth K (2009) Optical deconstruction of parkinsonian neural circuitry. Science 324: 354–359 19299587 10.1126/science.1167093 PMC6744370

[emmm201809575-bib-0051] Greenberg BD , Malone DA , Friehs GM , Rezai AR , Kubu CS , Malloy PF , Salloway SP , Okun MS , Goodman WK , Rasmussen SA (2006) Three‐year outcomes in deep brain stimulation for highly resistant obsessive‐compulsive disorder. Neuropsychopharmacology 31: 2384–2393 16855529 10.1038/sj.npp.1301165

[emmm201809575-bib-0052] Gubellini P , Salin P , Kerkerian‐Le Goff L , Baunez C (2009) Deep brain stimulation in neurological diseases and experimental models: from molecule to complex behavior. Prog Neurobiol 89: 79–123 19559747 10.1016/j.pneurobio.2009.06.003

[emmm201809575-bib-0053] Guo W , Nagappan G , Lu B (2018) Differential effects of transient and sustained activation of BDNF‐TrkB signaling. Dev Neurobiol 78: 647–659 29575722 10.1002/dneu.22592

[emmm201809575-bib-0054] Hahn PJ , McIntyre CC (2010) Modeling shifts in the rate and pattern of subthalamopallidal network activity during deep brain stimulation. J Comput Neurosci 28: 425–441 20309620 10.1007/s10827-010-0225-8 PMC2881193

[emmm201809575-bib-0055] Hamani C , MacHado DC , Hipólide DC , Dubiela FP , Suchecki D , MacEdo CE , Tescarollo F , Martins U , Covolan L , Nobrega JN (2012) Deep brain stimulation reverses anhedonic‐like behavior in a chronic model of depression: role of serotonin and brain derived neurotrophic factor. Biol Psychiatry 71: 30–35 22000731 10.1016/j.biopsych.2011.08.025 PMC5756076

[emmm201809575-bib-0056] Hamani C , Florence G , Heinsen H , Plantinga BR , Temel Y , Uludag K , Alho E , Teixeira MJ , Amaro E , Fonoff ET (2017) Subthalamic nucleus deep brain stimulation: basic concepts and novel perspectives. Eneuro 4: ENEURO.0140‐17.2017 10.1523/ENEURO.0140-17.2017 PMC5617209 28966978

[emmm201809575-bib-0057] Hanajima R , Ashby P , Lozano AM , Lang AE , Chen R (2004) Single pulse stimulation of the human subthalamic nucleus facilitates the motor cortex at short intervals. J Neurophysiol 92: 1937–1943 15152016 10.1152/jn.00239.2004

[emmm201809575-bib-0058] Hardenacke K , Kuhn J , Lenartz D , Maarouf M , Mai JK , Bartsch C , Freund HJ , Sturm V (2013) Stimulate or degenerate: deep brain stimulation of the nucleus basalis Meynert in Alzheimer dementia. World Neurosurg 80: e35–e43 10.1016/j.wneu.2012.12.005 23246738

[emmm201809575-bib-0059] Hartmann M , Heumann R , Lessmann V (2001) Synaptic secretion of BDNF after high‐frequency stimulation of glutamatergic synapses. EMBO J 20: 5887–5897 11689429 10.1093/emboj/20.21.5887 PMC125691

[emmm201809575-bib-0060] Hartmann CJ , Wojtecki L , Vesper J , Volkmann J , Groiss SJ , Schnitzler A , Südmeyer M (2015) Long‐term evaluation of impedance levels and clinical development in subthalamic deep brain stimulation for Parkinson's disease. Parkinsonism Relat Disord 21: 1247–1250 26234953 10.1016/j.parkreldis.2015.07.019

[emmm201809575-bib-0061] Hashimoto T , Elder CM , Okun MS , Patrick SK , Vitek JL (2003) Stimulation of the subthalamic nucleus changes the firing pattern of pallidal neurons. J Neurosci 23: 1916–1923 12629196 10.1523/JNEUROSCI.23-05-01916.2003 PMC6741976

[emmm201809575-bib-0062] Hassler R (1972) Sagittal thalamotomy for relief of motor disorders in cases of double athetosis and cerebral palsy. Confin Neurol 34: 18–28 4579790

[emmm201809575-bib-0063] Herron JA , Thompson MC , Brown T , Chizeck HJ , Ojemann JG , Ko AL (2017) Chronic electrocorticography for sensing movement intention and closed‐loop deep brain stimulation with wearable sensors in an essential tremor patient. J Neurosurg 127: 580–587 27858575 10.3171/2016.8.JNS16536

[emmm201809575-bib-0064] Hescham S , Lim LW , Jahanshahi A , Blokland A , Temel Y (2013) Deep brain stimulation in dementia‐related disorders. Neurosci Biobehav Rev 37: 2666–2675 24060532 10.1016/j.neubiorev.2013.09.002

[emmm201809575-bib-0065] Hilker R , Voges J , Ghaemi M , Lehrke R , Rudolf J , Koulousakis A , Herholz K , Wienhard K , Sturm V , Heiss W‐D (2003) Deep brain stimulation of the subthalamic nucleus does not increase the striatal dopamine concentration in parkinsonian humans. Mov Disord 18: 41–48 12518299 10.1002/mds.10297

[emmm201809575-bib-0066] Hoang KB , Cassar IR , Grill WM , Turner DA (2017) Biomarkers and stimulation algorithms for adaptive brain stimulation. Front Neurosci 11: 564 29066947 10.3389/fnins.2017.00564 PMC5641319

[emmm201809575-bib-0067] Holsheimer J , Dijkstra EA , Demeulemeester H , Nuttin B (2000) Chronaxie calculated from current‐duration and voltage‐duration data. J Neurosci Methods 97: 45–50 10771074 10.1016/s0165-0270(00)00163-1

[emmm201809575-bib-0068] Holtzheimer PE , Mayberg HS (2011) Deep brain stimulation for psychiatric disorders. Annu Rev Neurosci 34: 289–307 21692660 10.1146/annurev-neuro-061010-113638 PMC4413475

[emmm201809575-bib-0069] Holtzheimer PE , Husain MM , Lisanby SH , Taylor SF , Whitworth LA , Mcclintock S , Slavin KV , Honey CR , Neimat JS , Henderson JM *et al* (2017) Subcallosal cingulate deep brain stimulation for treatment‐resistant depression: a multisite, randomised, sham‐controlled trial. Lancet Psychiatry 4: 839–849 28988904 10.1016/S2215-0366(17)30371-1

[emmm201809575-bib-0070] Horn A , Reich M , Vorwerk J , Li N , Wenzel G , Fang Q , Schmitz‐Hübsch T , Nickl R , Kupsch A , Volkmann J *et al* (2017) Connectivity predicts deep brain stimulation outcome in Parkinson disease. Ann Neurol 4: 839–849 10.1002/ana.24974 PMC5880678 28586141

[emmm201809575-bib-0071] Hoyer C , Sartorius A (2012) Long‐term course of brain‐derived neurotrophic factor serum levels in a patient treated with deep brain stimulation of the lateral habenula. Neuropsychobiology 65: 147–152 22378223 10.1159/000335243

[emmm201809575-bib-0072] Hubble JP , Busenbark KL , Wilkinson S , Penn RD , Lyons K , Koller WC (1996) Deep brain stimulation for essential tremor. Neurology 46: 1150–1153 8780109 10.1212/wnl.46.4.1150

[emmm201809575-bib-0073] Huff W , Lenartz D , Schormann M , Lee SH , Kuhn J , Koulousakis A , Mai J , Daumann J , Maarouf M , Klosterkotter J *et al* (2010) Unilateral deep brain stimulation of the nucleus accumbens in patients with treatment‐resistant obsessive‐compulsive disorder: outcomes after one year. Clin Neurol Neurosurg 112: 137–143 20006424 10.1016/j.clineuro.2009.11.006

[emmm201809575-bib-0074] Humphries MD , Gurney K (2012) Network effects of subthalamic deep brain stimulation drive a unique mixture of responses in basal ganglia output. Eur J Neurosci 36: 2240–2251 22805068 10.1111/j.1460-9568.2012.08085.x

[emmm201809575-bib-0075] Huys D , Bartsch C , Koester P , Lenartz D , Maarouf M , Daumann J , Mai JK , Klosterkötter J , Hunsche S , Visser‐Vandewalle V *et al* (2016) Motor improvement and emotional stabilization in patients with Tourette syndrome after deep brain stimulation of the ventral anterior and ventrolateral motor part of the thalamus. Biol Psychiatry 79: 392–401 25034948 10.1016/j.biopsych.2014.05.014

[emmm201809575-bib-0076] Jahanshahi A , Schönfeld LM , Lemmens E , Hendrix S , Temel Y (2014) *In vitro* and *in vivo* neuronal electrotaxis: a potential mechanism for restoration? Mol Neurobiol 49: 1005–1016 24243342 10.1007/s12035-013-8575-7

[emmm201809575-bib-0077] Jiménez‐Sánchez L , Castañé A , Pérez‐Caballero L , Grifoll‐Escoda M , López‐Gil X , Campa L , Galofré M , Berrocoso E , Adell A (2016) Activation of AMPA receptors mediates the antidepressant action of deep brain stimulation of the infralimbic prefrontal cortex. Cereb Cortex 26: 2778–2789 26088969 10.1093/cercor/bhv133

[emmm201809575-bib-0078] Johnson LA , Vitek JL (2011) Deep brain stimulation: mechanisms of action In Youmans and Winn neurological surgery, Richard Winn H (ed.), Vol. 91, 6th edn, pp. 635–646. New York, NY: Elsevier

[emmm201809575-bib-0079] Kahan J , Urner M , Moran R , Flandin G , Marreiros A , Mancini L , White M , Thornton J , Yousry T , Zrinzo L *et al* (2014) Resting state functional MRI in Parkinson's disease: the impact of deep brain stimulation on ‘effective’ connectivity. Brain 137: 1130–1144 24566670 10.1093/brain/awu027 PMC3959559

[emmm201809575-bib-0080] Kalén P , Strecker RE , Rosengren E , Björklund A (1989) Regulation of striatal serotonin release by the lateral habenula‐dorsal raphe pathway in the rat as demonstrated by *in vivo* microdialysis: role of excitatory amino acids and GABA. Brain Res 492: 187–202 2473826 10.1016/0006-8993(89)90901-3

[emmm201809575-bib-0081] Kalia LV , Kalia SK , McLean PJ , Lozano AM , Lang AE (2013) α‐synuclein oligomers and clinical implications for Parkinson disease. Ann Neurol 137: 1130–1144 10.1002/ana.23746 PMC3608838 23225525

[emmm201809575-bib-0082] Kim SJ , Udupa K , Ni Z , Moro E , Gunraj C , Mazzella F , Lozano AM , Hodaie M , Lang AE , Chen R (2015) Effects of subthalamic nucleus stimulation on motor cortex excitability in Parkinson's disease. Neurology 119: 2513–2518 10.1212/WNL.0000000000001806 PMC4534071 26156511

[emmm201809575-bib-0083] Kringelbach ML , Jenkinson N , Green AL , Owen SLF , Hansen PC , Cornelissen PL , Holliday IE , Stein JF , Aziz TZ (2007a) Deep brain stimulation for chronic pain investigated with magnetoencephalography. NeuroReport 18: 223–228 17314661 10.1097/WNR.0b013e328010dc3d

[emmm201809575-bib-0084] Kringelbach ML , Jenkinson N , Owen SLF , Aziz TZ (2007b) Translational principles of deep brain stimulation. Nat Rev Neurosci 8: 623–635 17637800 10.1038/nrn2196

[emmm201809575-bib-0085] Kühn AA , Kupsch A , Schneider GH , Brown P (2006) Reduction in subthalamic 8‐35 Hz oscillatory activity correlates with clinical improvement in Parkinson's disease. Eur J Neurosci 23: 1956–1960 16623853 10.1111/j.1460-9568.2006.04717.x

[emmm201809575-bib-0086] Kuhn J , Lenartz D , Mai JK , Huff W , Lee S‐H , Koulousakis A , Klosterkoetter J , Sturm V (2007) Deep brain stimulation of the nucleus accumbens and the internal capsule in therapeutically refractory Tourette‐syndrome. J Neurol 254: 963–965 17410328 10.1007/s00415-006-0404-8

[emmm201809575-bib-0087] Kumar R , Dagher A , Hutchison WD , Lang AE , Lozano AM (1999) Globus pallidus deep brain stimulation for generalized dystonia: clinical and PET investigation. Neurology 53: 871–874 10489059 10.1212/wnl.53.4.871

[emmm201809575-bib-0088] Kuncel AM , Grill WM (2004) Selection of stimulus parameters for deep brain stimulation. Clin Neurophysiol 115: 2431–2441 15465430 10.1016/j.clinph.2004.05.031

[emmm201809575-bib-0089] Kupsch A , Tagliati M , Vidailhet M , Aziz T , Krack P , Moro E , Krauss JK (2011) Early postoperative management of DBS in dystonia: programming, response to stimulation, adverse events, medication changes, evaluations, and troubleshooting. Mov Disord 1: S37–S53 10.1002/mds.23624 21692111

[emmm201809575-bib-0090] Laxton AW , Tang‐Wai DF , McAndrews MP , Zumsteg D , Wennberg R , Keren R , Wherrett J , Naglie G , Hamani C , Smith GS *et al* (2010) A phase I trial of deep brain stimulation of memory circuits in Alzheimer's disease. Ann Neurol 68: 521–534 20687206 10.1002/ana.22089

[emmm201809575-bib-0091] Leal G , Comprido D , Duarte CB (2014) BDNF‐induced local protein synthesis and synaptic plasticity. Neuropharmacology 76: 639–656 23602987 10.1016/j.neuropharm.2013.04.005

[emmm201809575-bib-0092] Lee KH , Blaha CD , Harris BT , Cooper S , Hitti FL , Leiter JC , Roberts DW , Kim U (2006) Dopamine efflux in the rat striatum evoked by electrical stimulation of the subthalamic nucleus: potential mechanism of action in Parkinson's disease. Eur J Neurosci 23: 1005–1014 16519665 10.1111/j.1460-9568.2006.04638.x

[emmm201809575-bib-0093] Leong SL , De Ridder D , Vanneste S , Sutherland W , Ross S , Manning P (2018) High definition transcranial pink noise stimulation of anterior cingulate cortex on food craving: an explorative study. Appetite 120: 673–678 29079475 10.1016/j.appet.2017.10.034

[emmm201809575-bib-0094] Lhommée E , Wojtecki L , Czernecki V , Witt K , Maier F , Tonder L , Timmermann L , Hälbig TD , Pineau F , Durif F *et al* (2018) Behavioural outcomes of subthalamic stimulation and medical therapy versus medical therapy alone for Parkinson's disease with early motor complications (EARLYSTIM trial): secondary analysis of an open‐label randomised trial. Lancet Neurol 17: 223–231 29452685 10.1016/S1474-4422(18)30035-8

[emmm201809575-bib-0095] Li S , Arbuthnott GW , Jutras MJ , Goldberg JA , Jaeger D (2007) Resonant antidromic cortical circuit activation as a consequence of high‐frequency subthalamic deep‐brain stimulation. J Neurophysiol 98: 3525–3537 17928554 10.1152/jn.00808.2007

[emmm201809575-bib-0096] Lipsman N , Woodside DB , Giacobbe P , Hamani C , Carter JC , Norwood SJ , Sutandar K , Staab R , Elias G , Lyman CH *et al* (2013) Subcallosal cingulate deep brain stimulation for treatment‐refractory anorexia nervosa: a phase 1 pilot trial. Lancet 381: 1361–1370 23473846 10.1016/S0140-6736(12)62188-6

[emmm201809575-bib-0097] Little S , Brown P (2014) The functional role of beta oscillations in Parkinson's disease. Parkinsonism Relat Disord 20(Suppl 1): 44–48 10.1016/S1353-8020(13)70013-0 24262186

[emmm201809575-bib-0098] Little S , Beudel M , Zrinzo L , Foltynie T , Limousin P , Hariz M , Neal S , Cheeran B , Cagnan H , Gratwicke J *et al* (2016) Bilateral adaptive deep brain stimulation is effective in Parkinson's disease. J Neurol Neurosurg Psychiatry 87: 717–721 26424898 10.1136/jnnp-2015-310972 PMC4941128

[emmm201809575-bib-0099] Lozano AM , Eltahawy H (2004) Chapter 78: How does DBS work? Suppl Clin Neurophysiol 57: 733–736 16106676 10.1016/s1567-424x(09)70414-3

[emmm201809575-bib-0100] Lozano AM , Lipsman N (2013) Probing and regulating dysfunctional circuits using deep brain stimulation. Neuron 77: 406–421 23395370 10.1016/j.neuron.2013.01.020

[emmm201809575-bib-0101] Lozano AM , Fosdick L , Chakravarty MM , Leoutsakos J‐M , Munro C , Oh E , Drake KE , Lyman CH , Rosenberg PB , Anderson WS *et al* (2016) A phase II study of fornix deep brain stimulation in mild Alzheimer's disease. J Alzheimers Dis 54: 777–787 27567810 10.3233/JAD-160017 PMC5026133

[emmm201809575-bib-0102] Magarios‐Ascone C , Pazo JH , Macadar O , Buo W (2002) High‐frequency stimulation of the subthalamic nucleus silences subthalamic neurons: a possible cellular mechanism in Parkinson's disease. Neuroscience 115: 1109–1117 12453483 10.1016/s0306-4522(02)00538-9

[emmm201809575-bib-0103] Mann A , Gondard E , Tampellini D , Milsted JAT , Marillac D , Hamani C , Kalia SK , Lozano AM (2017) Chronic deep brain stimulation in an Alzheimer's disease mouse model enhances memory and reduces pathological hallmarks. Brain Stimul 11: 435–444 29246746 10.1016/j.brs.2017.11.012

[emmm201809575-bib-0104] McIntyre CC , Hahn PJ (2010) Network perspectives on the mechanisms of deep brain stimulation. Neurobiol Dis 38: 329–337 19804831 10.1016/j.nbd.2009.09.022 PMC2862840

[emmm201809575-bib-0105] McIntyre CC , Anderson RW (2016) Deep brain stimulation mechanisms: the control of network activity via neurochemistry modulation. J Neurochem 139: 338–345 27273305 10.1111/jnc.13649 PMC5358920

[emmm201809575-bib-0106] Meissner W , Reum T , Paul G , Harnack D , Sohr R , Morgenstern R , Kupsch A (2001) Striatal dopaminergic metabolism is increased by deep brain stimulation of the subthalamic nucleus in 6‐hydroxydopamine lesioned rats. Neurosci Lett 303: 165–168 11323111 10.1016/s0304-3940(01)01758-x

[emmm201809575-bib-0107] Meissner W , Harnack D , Paul G , Reum T , Sohr R , Morgenstern R , Kupsch A (2002) Deep brain stimulation of subthalamic neurons increases striatal dopamine metabolism and induces contralateral circling in freely moving 6‐hydroxydopamine‐lesioned rats. Neurosci Lett 328: 105–108 12133566 10.1016/s0304-3940(02)00463-9

[emmm201809575-bib-0108] Meissner W , Leblois A , Hansel D , Bioulac B , Gross CE , Benazzouz A , Boraud T (2005) Subthalamic high frequency stimulation resets subthalamic firing and reduces abnormal oscillations. Brain 128: 2372–2382 16123144 10.1093/brain/awh616

[emmm201809575-bib-0109] Melon C , Chassain C , Bielicki G , Renou J‐P , Kerkerian‐Le Goff L , Salin P , Durif F (2015) Progressive brain metabolic changes under deep brain stimulation of subthalamic nucleus in parkinsonian rats. J Neurochem 132: 703–712 25533782 10.1111/jnc.13015

[emmm201809575-bib-0110] Min H‐K , Ross EK , Jo HJ , Cho S , Settell ML , Jeong JH , Duffy PS , Chang S‐Y , Bennet KE , Blaha CD *et al* (2016) Dopamine release in the nonhuman primate caudate and putamen depends upon site of stimulation in the subthalamic nucleus. J Neurosci 36: 6022–6029 27251623 10.1523/JNEUROSCI.0403-16.2016 PMC4887566

[emmm201809575-bib-0111] Miocinovic S (2006) Computational analysis of subthalamic nucleus and lenticular fasciculus activation during therapeutic deep brain stimulation. J Neurophysiol 96: 1569–1580 16738214 10.1152/jn.00305.2006

[emmm201809575-bib-0112] Mirzadeh Z , Bari A , Lozano AM (2016) The rationale for deep brain stimulation in Alzheimer's disease. J Neural Transm 123: 775–783 26443701 10.1007/s00702-015-1462-9

[emmm201809575-bib-0113] Mohammadi A , Mehdizadeh AR (2016) Deep brain stimulation and gene expression alterations in Parkinson's disease. J Biomed Phys Eng 6: 47–50 27672624 PMC5022754

[emmm201809575-bib-0114] Mohseni HR , Smith PP , Parsons CE , Young KS , Hyam JA , Stein A , Stein JF , Green AL , Aziz TZ , Kringelbach ML (2012) MEG can map short and long‐term changes in brain activity following deep brain stimulation for chronic pain. PLoS One 7: e37993 22675503 10.1371/journal.pone.0037993 PMC3366994

[emmm201809575-bib-0115] Montgomery Jr EB , Gale JT (2008) Mechanisms of action of deep brain stimulation (DBS). Neurosci Biobehav Rev 32: 388–407 17706780 10.1016/j.neubiorev.2007.06.003

[emmm201809575-bib-0116] Moran A , Stein E , Tischler H , Belelovsky K , Bar‐Gad I (2011) Dynamic stereotypic responses of basal ganglia neurons to subthalamic nucleus high‐frequency stimulation in the parkinsonian primate. Front Syst Neurosci 5: 21 21559345 10.3389/fnsys.2011.00021 PMC3085177

[emmm201809575-bib-0117] Moro E , LeReun C , Krauss JK , Albanese A , Lin JP , Walleser Autiero S , Brionne TC , Vidailhet M (2017) Efficacy of pallidal stimulation in isolated dystonia: a systematic review and meta‐analysis. Eur J Neurol 24: 552–560 28186378 10.1111/ene.13255 PMC5763380

[emmm201809575-bib-0118] Moss F , Ward LM , Sannita WG (2004) Stochastic resonance and sensory information processing: a tutorial and review of application. Clin Neurophysiol 115: 267–281 14744566 10.1016/j.clinph.2003.09.014

[emmm201809575-bib-0119] Müller U , Sturm V , Voges J , Heinze H‐J , Galazky I , Büntjen L , Heldmann M , Frodl T , Steiner J , Bogerts B (2016) Nucleus accumbens deep brain stimulation for alcohol addiction – safety and clinical long‐term results of a pilot trial. Pharmacopsychiatry 49: 170–173 27145161 10.1055/s-0042-104507

[emmm201809575-bib-0120] Murer MG , Pazo JH (1993) Circling behaviour induced by activation of GABAA receptors in the subthalamic nucleus. NeuroReport 4: 1219–1222 8219017 10.1097/00001756-199309000-00002

[emmm201809575-bib-0121] Musacchio T , Rebenstorff M , Fluri F , Brotchie JM , Volkmann J , Koprich JB , Ip CW (2017) Subthalamic nucleus deep brain stimulation is neuroprotective in the A53T α‐synuclein Parkinson's disease rat model. Ann Neurol 81: 825–836 28470693 10.1002/ana.24947 PMC5519923

[emmm201809575-bib-0122] Nambu A , Takada M , Inase M , Tokuno H (1996) Dual somatotopical representations in the primate subthalamic nucleus: evidence for ordered but reversed body‐map transformations from the primary motor cortex and the supplementary motor area. J Neurosci 16: 2671–2683 8786443 10.1523/JNEUROSCI.16-08-02671.1996 PMC6578767

[emmm201809575-bib-0123] Navailles S , Benazzouz A , Bioulac B , Gross C , De Deurwaerdère P , De Deurwaerdere P , De Deurwaerdère P (2010) High‐frequency stimulation of the subthalamic nucleus and L‐3,4‐dihydroxyphenylalanine inhibit *in vivo* serotonin release in the prefrontal cortex and hippocampus in a rat model of Parkinson's disease. J Neurosci 30: 2356–2364 20147561 10.1523/JNEUROSCI.5031-09.2010 PMC6634027

[emmm201809575-bib-0124] Nimura T , Yamaguchi K , Ando T , Shibuya S , Oikawa T , Nakagawa A , Shirane R , Itoh M , Tominaga T (2005) Attenuation of fluctuating striatal synaptic dopamine levels in patients with Parkinson disease in response to subthalamic nucleus stimulation: a positron emission tomography study. J Neurosurg 103: 968–973 16381182 10.3171/jns.2005.103.6.0968

[emmm201809575-bib-0125] Nowak LG , Bullier J (1998) Axons, but not cell bodies, are activated by electrical stimulation in cortical gray matter. I. Evidence from chronaxie measurements. Exp Brain Res 118: 477–488 9504843 10.1007/s002210050304

[emmm201809575-bib-0126] Nuttin B , Gielen F , Van Kuyck K , Wu H , Luyten L , Welkenhuysen M , Brionne TC , Gabriëls L (2013) Targeting bed nucleus of the stria terminalis for severe obsessive‐compulsive disorder: more unexpected lead placement in obsessive‐compulsive disorder than in surgery for movement disorders. World Neurosurg 80: 11–16 10.1016/j.wneu.2012.12.029 23268197

[emmm201809575-bib-0127] Owen SLF , Green AL , Stein JF , Aziz TZ (2006) Deep brain stimulation for the alleviation of post‐stroke neuropathic pain. Pain 120: 202–206 16359796 10.1016/j.pain.2005.09.035

[emmm201809575-bib-0128] Perlmutter JS , Mink JW (2006) Deep brain stimulation. Annu Rev Neurosci 29: 229–257 16776585 10.1146/annurev.neuro.29.051605.112824 PMC4518728

[emmm201809575-bib-0129] Picillo M , Lozano AM , Kou N , Puppi Munhoz R , Fasano A (2016) Programming deep brain stimulation for Parkinson's disease: the Toronto western hospital algorithms. Brain Stimul 9: 425–437 26968806 10.1016/j.brs.2016.02.004

[emmm201809575-bib-0130] Pienaar IS , Lee CH , Elson JL , McGuinness L , Gentleman SM , Kalaria RN , Dexter DT (2015) Deep‐brain stimulation associates with improved microvascular integrity in the subthalamic nucleus in Parkinson's disease. Neurobiol Dis 74: 392–405 25533682 10.1016/j.nbd.2014.12.006

[emmm201809575-bib-0131] Pierantozzi M , Palmieri MG , Mazzone P , Marciani MG , Rossini PM , Stefani A , Giacomini P , Peppe A , Stanzione P (2002) Deep brain stimulation of both subthalamic nucleus and internal globus pallidus restores intracortical inhibition in Parkinson's disease paralleling apomorphine effects: a paired magnetic stimulation study. Clin Neurophysiol 113: 108–113 11801431 10.1016/s1388-2457(01)00694-0

[emmm201809575-bib-0132] Ranck JB (1975) Which elements are excited in electrical stimulation of mammalian central nervous system: a review. Brain Res 21: 417–440 10.1016/0006-8993(75)90364-9 1102064

[emmm201809575-bib-0133] Rauskolb S , Zagrebelsky M , Dreznjak A , Deogracias R , Matsumoto T , Wiese S , Erne B , Sendtner M , Schaeren‐Wiemers N , Korte M *et al* (2010) Global deprivation of brain‐derived neurotrophic factor in the CNS reveals an area‐specific requirement for dendritic growth. J Neurosci 30: 1739–1749 20130183 10.1523/JNEUROSCI.5100-09.2010 PMC6633992

[emmm201809575-bib-0134] Reddy GD , Lozano AM (2017) Postmortem studies of deep brain stimulation for Parkinson's disease: a systematic review of the literature. Cell Tissue Res 373: 287–295 28836072 10.1007/s00441-017-2672-2

[emmm201809575-bib-0135] Remy P , Doder M , Lees A , Turjanski N , Brooks D (2005) Depression in Parkinson's disease: loss of dopamine and noradrenaline innervation in the limbic system. Brain 128: 1314–1322 15716302 10.1093/brain/awh445

[emmm201809575-bib-0136] Russo M , Cousins MJ , Brooker C , Taylor N , Boesel T , Sullivan R , Poree L , Shariati NH , Hanson E , Parker J (2018) Effective relief of pain and associated symptoms with closed‐loop spinal cord stimulation system: preliminary results of the Avalon study. Neuromodulation 21: 38–47 28922517 10.1111/ner.12684

[emmm201809575-bib-0137] Sankar T , Lipsman N , Lozano AM (2014) Deep brain stimulation for disorders of memory and cognition. Neurotherapeutics 11: 527–534 24777384 10.1007/s13311-014-0275-0 PMC4121440

[emmm201809575-bib-0138] Sankar T , Chakravarty MM , Bescos A , Lara M , Obuchi T , Laxton AW , McAndrews MP , Tang‐Wai DF , Workman CI , Smith GS *et al* (2015) Deep brain stimulation influences brain structure in Alzheimer's disease. Brain Stimul 8: 645–654 25814404 10.1016/j.brs.2014.11.020 PMC5659851

[emmm201809575-bib-0139] Sartorius A , Kiening KL , Kirsch P , von Gall CC , Haberkorn U , Unterberg AW , Henn FA , Meyer‐Lindenberg A (2010) Remission of major depression under deep brain stimulation of the lateral habenula in a therapy‐refractory patient. Biol Psychiatry 67: e9–e11 19846068 10.1016/j.biopsych.2009.08.027

[emmm201809575-bib-0140] Sasaki T , Matsuki N , Ikegaya Y (2011) Action‐potential modulation during axonal conduction. Science 331: 599–601 21292979 10.1126/science.1197598

[emmm201809575-bib-0141] Sauleau P , Drapier S , Duprez J , Houvenaghel JF , Dondaine T , Haegelen C , Drapier D , Jannin P , Robert G , Le Jeune F *et al* (2016) Weight gain following pallidal deep brain stimulation: a PET Study. PLoS One 11: e0153438 27070317 10.1371/journal.pone.0153438 PMC4829218

[emmm201809575-bib-0142] Schlaepfer TE , Bewernick BH , Kayser S , Mädler B , Coenen VA (2013) Rapid effects of deep brain stimulation for treatment‐resistant major depression. Biol Psychiatry 73: 1204–1212 23562618 10.1016/j.biopsych.2013.01.034

[emmm201809575-bib-0143] Schuepbach WMM , Rau J , Knudsen K , Volkmann J , Krack P , Timmermann L , Hälbig TD , Hesekamp H , Navarro SM , Meier N *et al* (2013) Neurostimulation for Parkinson's disease with early motor complications. N Engl J Med 368: 610–622 23406026 10.1056/NEJMoa1205158

[emmm201809575-bib-0144] Shen KZ , Zhu ZT , Munhall A , Johnson SW (2003) Synaptic plasticity in rat subthalamic nucleus induced by high‐frequency stimulation. Synapse 50: 314–319 14556236 10.1002/syn.10274

[emmm201809575-bib-0145] Shin DS , Samoilova M , Cotic M , Zhang L , Brotchie JM , Carlen PL (2007) High frequency stimulation or elevated K^+^ depresses neuronal activity in the rat entopeduncular nucleus. Neuroscience 149: 68–86 17826920 10.1016/j.neuroscience.2007.06.055

[emmm201809575-bib-0146] Shirota Y , Ohtsu H , Hamada M , Enomoto H , Ugawa Y (2013) Supplementary motor area stimulation for Parkinson disease: a randomized controlled study. Neurology 80: 1400–1405 23516319 10.1212/WNL.0b013e31828c2f66

[emmm201809575-bib-0147] Silberstein P , Kühn AA , Kupsch A , Trottenberg T , Krauss JK , Wöhrle JC , Mazzone P , Insola A , Di Lazzaro V , Oliviero A *et al* (2003) Patterning of globus pallidus local field potentials differs between Parkinson's disease and dystonia. Brain 126: 2597–2608 12937079 10.1093/brain/awg267

[emmm201809575-bib-0148] Song S , Song S , Cao C , Lin X , Li K , Sava V , Sanchez‐Ramos J (2013) Hippocampal neurogenesis and the brain repair response to brief stereotaxic insertion of a microneedle. Stem Cells Int 2013: 205878 23554817 10.1155/2013/205878 PMC3608357

[emmm201809575-bib-0149] Soreq L , Bergman H , Israel Z , Soreq H (2013) Deep brain stimulation modulates nonsense‐mediated RNA decay in Parkinson's patients leukocytes. BMC Genom 14: 478 10.1186/1471-2164-14-478 PMC3723527 23865419

[emmm201809575-bib-0150] Spiegel EA , Wycis HT (1952) Thalamotomy and pallidotomy for treatment of choreic movements. Acta Neurochir (Wien) 2: 417–422 12976060 10.1007/BF01405833

[emmm201809575-bib-0151] Spieles‐Engemann AL , Behbehani MM , Collier TJ , Wohlgenant SL , Steece‐Collier K , Paumier K , Daley BF , Gombash S , Madhavan L , Mandybur GT *et al* (2010) Stimulation of the rat subthalamic nucleus is neuroprotective following significant nigral dopamine neuron loss. Neurobiol Dis 39: 105–115 20307668 10.1016/j.nbd.2010.03.009 PMC2879040

[emmm201809575-bib-0152] Stefani A , Fedele E , Vitek J , Pierantozzi M , Galati S , Marzetti S , Peppe A , Bassi MS , Bernardi G , Stanzione P (2011) The clinical efficacy of L‐DOPA and STN‐DBS share a common marker: reduced GABA content in the motor thalamus. Cell Death Dis 2: e154 21544093 10.1038/cddis.2011.35 PMC3122115

[emmm201809575-bib-0153] Stone SSD , Teixeira CM , DeVito LM , Zaslavsky K , Josselyn SA , Lozano AM , Frankland PW (2011) Stimulation of entorhinal cortex promotes adult neurogenesis and facilitates spatial memory. J Neurosci 31: 13469–13484 21940440 10.1523/JNEUROSCI.3100-11.2011 PMC6623309

[emmm201809575-bib-0154] Strafella AP , Sadikot AF , Dagher A (2003) Subthalamic deep brain stimulation does not induce striatal dopamine release in Parkinson's disease. NeuroReport 14: 1287–1289 12824777 10.1097/00001756-200307010-00020

[emmm201809575-bib-0155] Tai C‐H (2003) Electrophysiological and metabolic evidence that high‐frequency stimulation of the subthalamic nucleus bridles neuronal activity in the subthalamic nucleus and the substantia nigra reticulata. FASEB J 17: 1820–1830 14519661 10.1096/fj.03-0163com

[emmm201809575-bib-0156] Tan SKH , Hartung H , Schievink S , Sharp T , Temel Y (2013) High‐frequency stimulation of the substantia nigra induces serotonin‐dependent depression‐like behavior in animal models. Biol Psychiatry 73: e1–e3 22939751 10.1016/j.biopsych.2012.07.032

[emmm201809575-bib-0157] Tawfik VL , Chang SY , Hitti FL , Roberts DW , Leiter JC , Jovanovic S , Lee KH (2010) Deep brain stimulation results in local glutamate and adenosine release: investigation into the role of astrocytes. Neurosurgery 67: 367–375 20644423 10.1227/01.NEU.0000371988.73620.4C PMC2919357

[emmm201809575-bib-0158] Temel Y , Visser‐Vandewalle V , Kaplan S , Kozan R , Daemen MARC , Blokland A , Schmitz C , Steinbusch HWM (2006) Protection of nigral cell death by bilateral subthalamic nucleus stimulation. Brain Res 1120: 100–105 16999940 10.1016/j.brainres.2006.08.082

[emmm201809575-bib-0159] Temel Y , Boothman LJ , Blokland A , Magill PJ , Steinbusch HWM , Visser‐Vandewalle V , Sharp T (2007) Inhibition of 5‐HT neuron activity and induction of depressive‐like behavior by high‐frequency stimulation of the subthalamic nucleus. Proc Natl Acad Sci USA 104: 17087–17092 17942692 10.1073/pnas.0704144104 PMC2040465

[emmm201809575-bib-0160] Temel Y , Tan S , Visser‐Vandewalle V , Sharp T (2009) Parkinson's disease, DBS and suicide: a role for serotonin. Brain 132: e126 19553275 10.1093/brain/awp150

[emmm201809575-bib-0161] Thobois S , Fraix V , Savasta M , Costes N , Pollak P , Mertens P , Koudsie A , Le Bars D , Benabid AL , Broussolle E (2003) Chronic subthalamic nucleus stimulation and striatal D2 dopamine receptors in Parkinson's disease–A [(11)C]‐raclopride PET study. J Neurol 250: 1219–1223 14586606 10.1007/s00415-003-0188-z

[emmm201809575-bib-0162] Torres‐Sanchez S , Perez‐Caballero L , Berrocoso E (2017) Cellular and molecular mechanisms triggered by deep brain stimulation in depression: a preclinical and clinical approach. Prog Neuropsychopharmacology Biol Psychiatry 73: 1–10 10.1016/j.pnpbp.2016.09.005 27644164

[emmm201809575-bib-0163] Torres‐Sanchez S , Perez‐Caballero L , Mico JA , Celada P , Berrocoso E (2018) Effect of deep brain stimulation of the ventromedial prefrontal cortex on the noradrenergic system in rats. Brain Stimul 11: 222–230 29074339 10.1016/j.brs.2017.10.003

[emmm201809575-bib-0164] Tronnier VM , Rasche D , Thorns V , Alvarez‐Fischer D , Münte TF , Zurowski B (2018) Massive weight loss following deep brain stimulation of the nucleus accumbens in a depressed woman. Neurocase 24: 49–53 29388475 10.1080/13554794.2018.1431678

[emmm201809575-bib-0165] Tsukahara T , Takeda M , Shimohama S , Ohara O , Hashimoto N (1995) Effects of brain‐derived neurotrophic factor on 1‐methyl‐4‐phenyl‐1,2,3,6‐tetrahydropyridine‐induced parkinsonism in monkeys. Neurosurgery 37: 733–739 8559303 10.1227/00006123-199510000-00018

[emmm201809575-bib-0166] Udupa K , Chen R (2015) The mechanisms of action of deep brain stimulation and ideas for the future development. Prog Neurobiol 133: 27–49 26296674 10.1016/j.pneurobio.2015.08.001

[emmm201809575-bib-0167] Van Dijk A , Mason O , Klompmakers AA , Feenstra MGP , Denys D (2011) J Neurosci Methods 202: 113–118 21565219 10.1016/j.jneumeth.2011.04.034

[emmm201809575-bib-0168] Van Dijk A , Klompmakers AA , Feenstra MGP , Denys D (2012) Deep brain stimulation of the accumbens increases dopamine, serotonin, and noradrenaline in the prefrontal cortex. J Neurochem 123: 897–903 23061486 10.1111/jnc.12054

[emmm201809575-bib-0169] Vedam‐Mai V , Gardner B , Okun MS , Siebzehnrubl FA , Kam M , Aponso P , Steindler DA , Yachnis AT , Neal D , Oliver BU *et al* (2014) Increased precursor cell proliferation after deep brain stimulation for Parkinson's disease: a human study. PLoS One 9: e88770 24594681 10.1371/journal.pone.0088770 PMC3940428

[emmm201809575-bib-0170] Walker RH , Moore C , Davies G , Dirling LB , Koch RJ , Meshul CK (2012) Effects of subthalamic nucleus lesions and stimulation upon corticostriatal afferents in the 6‐hydroxydopamine‐lesioned rat. PLoS One 7: e32919 22427909 10.1371/journal.pone.0032919 PMC3299711

[emmm201809575-bib-0171] Wallace BA , Ashkan K , Heise CE , Foote KD , Torres N , Mitrofanis J , Benabid AL (2007) Survival of midbrain dopaminergic cells after lesion or deep brain stimulation of the subthalamic nucleus in MPTP‐treated monkeys. Brain 130: 2129–2145 17584773 10.1093/brain/awm137

[emmm201809575-bib-0172] van Westen M , Rietveld E , Figee M , Denys D (2015) Clinical outcome and mechanisms of deep brain stimulation for obsessive‐compulsive disorder. Curr Behav Neurosci Rep 2: 41–48 26317062 10.1007/s40473-015-0036-3 PMC4544542

[emmm201809575-bib-0173] Whiting DM , Tomycz ND , Bailes J , de Jonge L , Lecoultre V , Wilent B , Alcindor D , Prostko ER , Cheng BC , Angle C *et al* (2013) Lateral hypothalamic area deep brain stimulation for refractory obesity: a pilot study with preliminary data on safety, body weight, and energy metabolism. J Neurosurg 119: 58–63 10.3171/2013.2.JNS12903 PMC5666570 23560573

[emmm201809575-bib-0174] Wojtecki L , Groiss SJ , Hartmann CJ , Elben S , Omlor S , Schnitzler A , Vesper J (2016) Deep brain stimulation in Huntington's disease—preliminary evidence on pathophysiology, efficacy and safety. Brain Sci 6: E38 27589813 10.3390/brainsci6030038 PMC5039467

[emmm201809575-bib-0175] Xia F , Yiu A , Stone SSD , Oh S , Lozano AM , Josselyn SA , Frankland PW (2017) Entorhinal cortical deep brain stimulation rescues memory deficits in both young and old mice genetically engineered to model Alzheimer's disease. Neuropsychopharmacology 42: 2493–2503 28540926 10.1038/npp.2017.100 PMC5686482

